# Proliferation of Human Cervical Cancer Cells Responds to Surface Properties of Bicomponent Polymer Coatings

**DOI:** 10.3390/nano15100716

**Published:** 2025-05-09

**Authors:** Emil Rosqvist, Erik Niemelä, Shujun Liang, John E. Eriksson, Xiaoju Wang, Jouko Peltonen

**Affiliations:** 1 Laboratory of Molecular Science and Engineering, Åbo Akademi University, Henriksgatan 2, 20500 Åbo, Finland; 2 Centre for Functional Materials, Laboratory of Cell Biology, Åbo Akademi University, Artillerigatan 6, 20520 Åbo, Finland; 3 Pharmaceutical Sciences Laboratory, Åbo Akademi University, Tykistökatu 6A, 20520 Åbo, Finland; 4 Laboratory of Natural Materials Technology, Åbo Akademi University, Henriksgatan 2, 20500 Åbo, Finland; 5 Turku Bioscience Centre, University of Turku and Åbo Akademi University, 20520 Åbo, Finland; 6 Euro-BioImaging ERIC, 20520 Åbo, Finland

**Keywords:** nanostructure, surface roughness, epithelial cell, fibroblast, cell growth, passive control, latex, atomic force microscopy (AFM), cell proliferation, cell–surface interaction

## Abstract

The proliferation of human cervical cancer (Hela) cells was investigated on a series of nanostructured polymer latex surfaces. The physico-chemical properties of the surfaces, composed of mixtures of polystyrene and acrylonitrile butadiene styrene dispersions, were precisely controlled in the nanoscale range by adjusting the mixing ratio of the components and thermal treatment. In addition, the proliferation response of HeLa cells was compared to that of human dermal fibroblast (HDF) cells. A low dispersive surface energy and peak or valley dominance (S_pk_/S_vk_) were observed to increase the proliferation yield of the Hela cells. The HDF cells were less influenced by the surface chemistry and showed improved proliferation on surfaces without dominant peak or valley features (S_pk_ and S_vk_). The observed changes in Hela cell behaviour underscored the critical role of material surface properties in influencing cellular responses, with more significant accumulation of nuclear patterning of filamentous actin (F-actin) on stiffer and smoother surfaces (e.g., borosilicate glass) due to higher mechanical stress. A more dynamic reorganisation of the cytoskeleton was observed for cells grown on polymer surfaces with moderate roughness and surface energy. These results emphasise the importance of characterising and tuning surface properties to accommodate the specific behaviours of different cell types.

## 1. Introduction

Surface properties affect interactions between cells and materials. These response-driving cues are provided by the surrounding cells, the surrounding materials, the extracellular matrix (ECM), and other cells, and the cells adapt to them accordingly [1]. Cell–surface interactions can be complex, and different cells may respond very differently to particular surface characteristics. This makes such studies challenging, yet highly intriguing. Cell–material interactions can drive and modulate cell attachment, viability, mobility, differentiation, and tumour formation. Detailed studies aim to develop models to explain the mechanisms behind the cellular responses and the disruption of cellular homeostasis [1]. An improved understanding of cellular responses would enable the development of new biomaterials tailored for specific applications and could pave the way for new materials for disease treatment strategies. As an example, the response of antigen-presenting cells, which are crucial, e.g., in implant healing, can be controlled by modifying topography and surface chemistry [2].

It is essential to consider both surface chemistry and topography and their combined effects when relating the biological responses to material surface properties. A large variety of different roughness parameters are available for versatile characterisation of a surface. Indeed, vastly different surface geometries can appear similar if using a few roughness parameters, which do not provide a comprehensive description of the surface. Besides the commonly used average roughness (S_a_) or RMS roughness (root mean square, S_q_) parameters for describing height roughness, other useful parameters are, among others, effective surface area, summit density, correlation lengths, and functional surface parameters such as peak height and valley depth [3,4,5]. Indeed, using solely the S_a_ and S_q_ parameters is inadequate when studying particle–surface interactions [6].

Human tumours arise from normal cells accumulating mutations, which favour exponential growth and epithelial-to-mesenchymal transformation. This reshapes the microenvironment inside the tumour tissue, which in turn leads to changes in surface properties and tissue stiffness. The tumour environment induces inflammation as well as nutrient and oxygen deprivation, leading to activation of cancer-associated fibroblasts (CAFs). Normally, fibroblasts are vital in wound repair. However, CAFs are perpetually activated, leading to tissue fibrosis. Both the activated CAFs and the restructured fibrotic tissue promote tumour growth even further to a vicious self-promoting growth cycle [7]. Hela cells are one of the most widely used immortalised epithelial cell lines in cell studies [8,9]. In 3D cell cultures, Hela cells have the capability to undergo EMT and acquire invasive properties [10]. Furthermore, this cell type has also been observed to respond to surface properties. This has been observed in studies of the effect of surface modifications of poly(dimethylsiloxane) [8] and tissue-culture polystyrene—the latter by fluoropolymers and a blend of fluoropolymer and silica nanoparticles [8]. The adhesion of Hela cells has been observed to respond to stiffness and elastic modulus of soft hydrogel surfaces [11]. Roughness (measured by stylus profilometry), together with surface energy, has also been observed to drive cell attachment, growth, and spreading [1,12]. Similarly, chondrosarcoma cells have been observed to respond to the stiffness of crosslinked poly(L-lysine)/hyaluronan films [13]. The response of Hela cells to surface properties can be seen during metastasis and nanoparticle uptake [14,15]. Also, a stiffer matrix has been shown to induce nuclear localisation and expression of YAP/TAZ proteins [16]. Therefore, studying interactions of both Hela and fibroblast cells with different surfaces improves the understanding of cancer formation and disruptions in cellular homeostasis induced by variations in the microenvironment.

In this study, we investigated the cell viability and morphological response of Hela cells to a set of two-component latex polymer surfaces. The objective was to allow for a thorough analysis and comparison of the viability response of the Hela cells to the physico-chemical properties of the surfaces, that is, surface chemistry and surface topography. These latex surfaces were used for the possibility to tune their surface topography and high processability. Roll-to-roll methods can be used to process them into cell-study platforms with tuneable surface properties that offer the cells a more natural environment in 2.5D screening, or allow for new approaches to cell–surface interactions [17], and they can be processed into Surface-Enhanced Raman Spectroscopy active surfaces [18]. Characterisation of the surfaces with a substantial set of roughness parameters provides detailed information on such topographical features that are relevant to cell–surface interactions [4,19]. It was also of interest to compare the response of Hela cells to that of HDF cells on the same surfaces, as was performed in [20]. Herein, the analysis of topographical drivers of HDF viability was also deepened. In addition to the HDF viability, these surfaces have previously been observed to affect the *Staphylococcus aureus* phenotype and biofilm formation [21], as well as ARPE-19 cells [22]. Surface–protein interactions have also been studied [23]. Cell–surface interaction studies with several cell lines, both mammalian and bacterial cells, allow for the use of them in more complex environments where the growth of some cells needs to be enhanced and some impeded, such as implant materials. This is also essential for deepening the understanding of how different cells respond to the same surface properties.

## 2. Materials and Methods

### 2.1. Surfaces 

The surfaces used in this study were manufactured using two aqueous latex dispersions, one with a high glass transition temperature (T_g_) and the other with a low T_g_. The high-T_g_ latex (105 °C) was a dispersion of polystyrene (PS) particles (HPY83, Styron Europe GmbH, CH, Richterswil, Switzerland) with an average particle diameter of 130 nm. The low-T_g_ (8–10 °C) dispersion consisted of acrylonitrile butadiene styrene (ABS) copolymer particles (HPC26, DOW Europe GmbH, CH, Horgen, Switzerland) with an average particle diameter of 140 nm, as reported by the manufacturer. The solids contents of the high-T_g_ latex and the low-T_g_ latex dispersions were 47.9% and 54.5%. The surface films were manufactured by volumetric mixes of the PS and ABS dispersions, and the volumetric ratio of the PS was used to name the samples. The dispersions were drop-cast on borosilicate glass coverslip substrates with a radius of 13 mm (VWR, ECN 631-1577, Darmstadt, Germany) (30 µL per coverslip). These coverslips were also used as a reference for normalising the viability data.

The final nanostructured surface texture was obtained through irradiation of the latex-coated substrate 1 h after drop casting with an infrared (IR) heater (IRT systems, Hedson Technologies AB, Arlöv, Sweden). During the heat treatment, the PS particles in the film co-annealed and deformed. In addition, the relative particle size, mixing ratio, and film formation temperature of the components contributed to the final topography and surface chemistry [24,25]. The IR-treated samples were rinsed with water and ethanol and dried in ambient conditions. Good adhesion between the latex film and the glass surface was ensured by a 60 min heat treatment at 105 °C, i.e., above the boiling point of water but below the T_g_ of the polymer. It has been shown that non-ionic, hydrophilic sulphonate surfactant additives in the ABS are removed during IR thermal annealing [1].

These surfaces have been shown to be highly transparent for visible wavelengths (90%), even when coated with ultra-thin gold films (70%) [2]. This allowed for optical studies of the cells.

### 2.2. Cell Culture 

Hela and HDF cells were acquired from ATCC (Manassas, VA, USA). They were grown and maintained in DMEM medium (Sigma-Aldrich, St. Louis, MO, USA) with 10% foetal calf serum (BioClear, Wiltshire, UK), 2 mM L-glutamin, 100 U/mL penicillin and 100 µg/mL streptomycin at 37 °C in a 5% CO_2_/95% O_2_ and 90% RH atmosphere, and they were kept under sterile conditions in a cell culture incubator.

### 2.3. Atomic Force Microscopy

The topography of the surfaces was imaged with a Nanoscope V MultiMode 8 atomic force microscope (AFM; Bruker, Billerica, MA, USA). Images of 5 µm by 5 µm size and a resolution of 1024 by 1024 pixels were captured with NSG10 cantilevers with a nominal tip radius of 10 nm (NT-MDT, Apeldoorn, The Netherlands).

The image analysis software Scanning Probe Image Processor (SPIP) by Image Metrology (Lyngby, Denmark) was used for image analysis and calculation of the surface roughness parameter values. The captured topographic images of the latex surfaces were processed with the software’s Gaussian filter according to ISO 16610-21:2011 standards [26]. A 0-th degree LMS fit was also applied when necessary.

Several different roughness parameters were calculated from the AFM images. The RMS roughness (S_q_) measures vertical deviations from the mean plane as the root mean square average of all height points. The surface area ratio (S_dr_) is a measure of the roughness-induced increase in area compared to the projected flat plane. The autocorrelation length from the autocorrelation function (S_cl37_) is a measure of the lateral spacing between surface features, the definition being the length over which the correlation function reduces to 37%, i.e., 1/e, of its initial height at the origin [3,4]. Skewness (S_sk_) describes height asymmetry, i.e., the distribution of height values in relation to the mean height. A negative skewness represents a surface dominated by valleys (heights lower than the mean), while for a positive skewness, peaks dominate the height distribution. Kurtosis (S_ku_) describes the shape relative to the mean, with a normal distribution having a kurtosis of 3. A distribution with a kurtosis higher than 3 has tails that approach 0 more quickly than the normal distribution, while for a distribution with a kurtosis lower than 3, the tails approach 0 more slowly. The density of summits, S_ds_, is a measure of the number of local maxima per unit area. Similarly, functional parameters obtained from the bearing area curve are utilised. Parameters such as S_pk_ (the reduced peak height), which relates to the height of the peak region of the surface, S_vk_ (the reduced valley depth), which relates to the valley depth of the surface, and S_k_, which relates to the height of the core layer roughness S_k_ (the core roughness) can be read out from the material ratio curve as follows. The material ratio curve is a plot of the sum of the ratio of material intercept through a bearing plane, which is parallel to the mean plane, at each height of the surface plot. In the material ratio curve, a least mean square line (LMS) is fitted to 40% of the curve in such a way that the LMS line has the lowest decline (see Supplementary Material Figure S21). The S_k_ is the height difference between extrapolations of the LMS to 0% and 100% material ratios. Further, if horizontal lines are drawn from the top and bottom levels of the LMS curve, where these intersect the material ratio curve, so that the areas of the formed triangles are equal to the area between the horizontal line and the bearing area curve, the heights of the triangles give the S_pk_ and the S_vk_ [27]. These last three parameters have been observed to distinguish between surfaces that have a similar amplitude roughness [28] and have been useful in analysing the response of *S. aureus* to nanostructured surfaces [21].

### 2.4. Contact Angle Measurements and Surface Energy Determination

The equilibrium contact angles (CAs) were determined with a CAM200 goniometer (KSV Instruments Ltd., Helsinki, Finland). Three probe liquids were used, MilliQ water, diiodo methane (DIM) and ethylene glycol (EG), to allow for calculating the surface energy. The droplet volume was 2 µL (1.4 µL for DIM). Contact angles were determined from the captured images of the droplets using the instrument software, which utilised a Young–Laplace fitting method to the droplet silhouette curvature. The measured contact angle values were corrected for roughness using the S_dr_ parameter as described in [25]. Readers should be aware of the ongoing discussion of the applicability of the Wenzel equation [29,30,31].

Surface energies were calculated using the van Oss–Chaudhury–Good method. According to Fowkes, this the sum of all contributions of different intermolecular interactions, accounting for dispersive interaction forces (London forces), dipole–dipole interactions (Keesom), permanent-induced dipole interactions (Debye), hydrogen bonds, etc. Van Oss, Chaudhury, and Good proposed that this can be sufficiently approximated by considering van der Waals interactions and acid–base interactions [32,33]:

(1) $$\gamma_{i}=\gamma_{i}^{LW}+\gamma_{i}^{AB}.$$

The acid and base contributions, i.e., polar-positive and polar-negative characteristics, of a surface can be separated as

(2) $$\gamma_{i}^{AB}=2{\left(\gamma_{i}^{+}\gamma_{i}^{-}\right)}^{\frac{1}{2}}.$$

The $\gamma_{i}^{LW}$ components account for London, Keesom, and Debye forces, while $\gamma_{i}^{AB}$ accounts for polar interactions, such as hydrogen bonding as well as donor–acceptor pairs and electrophile–nucleophile interactions [32]. Surface energies were calculated using the van Oss–Chaudhury–Good method. The surface tension values of the probe liquids were those published by van Oss–Chaudhury–Good [5].

### 2.5. Light Microscopy for Observing Cell Proliferation

Both HDF and Hela cells were seeded at a density of 5∙10^5^ cells per glass coverslip or coated glass coverslip that had been placed in 24-well plates. Cell growth was conducted in complete DMEM cell medium. Samples were sterilised using 70% ethanol before cell plating. The cell growth was conducted as follows: First, the cells were allowed to adhere to the surfaces over a period of 4 h, after which the plates were placed in an automated Cell-IQ live cell imaging instrument (CM Technologies, Lieto, Finland) for a further period of 72 h. The total incubation time was thus 96 h. With the Cell-IQ, a 3 × 3 phase contrast image raster of the substrates was obtained for each well at six-hour intervals.

### 2.6. Crystal Violet Staining for Quantifying Cell Growth

After the incubation, both HDF and Hela cells were washed with phosphate-buffered saline (PBS), and the nuclei were stained using 0.2% crystal violet dye (CV) in a 2% ethanol solution. Excess dye was rinsed off with repeated MilliQ water washing cycles, and then air-dried at RT. After drying, the CV was re-solubilised in 2% sodium dodecyl sulphate (SDS) and the absorbance of each well was determined at 570 nm using a HIDEX micro-plate reader (Hidex, Turku, Finland). To give an end-point measurement of cell growth on different materials of interest, the absorbance of each sample was normalised to the mean value of the glass coverslip samples measured in parallel. Here, this is called the ‘viability’ of the cells.

### 2.7. Cell Viability and Cellular Morphology

A total of 5 × 10^5^ Hela cells were seeded onto glass coverslips or glass coverslips coated with the different latex blends. The cells were cultured with complete medium in 24-well plates. The viability of the Hela cells was evaluated for 96 h using a live/dead cell assay. At two time points, 24 h and 96 h, the Hela cells were stained with 0.5 µm Calcein-AM/1.6 µm EthD-III in PBS and incubated at 37 °C and 5% CO_2_ for 1 h. After this, the cells were imaged with fluorescence microscopy using a Zeiss Axio Vert.A1 microscope (Zeiss, Oberkochen, Germany) and further processed with Fiji ImageJ software (v. 1.54f) [34].

To better understand the cell–material interaction, a total of 5 × 10^5^ Hela cells (per coverslip) were seeded onto glass coverslips and selected latex coatings. After 24 h of incubation, Hela cells were stained to observe vimentin, F-actin, and the nuclei. A brief description of the staining protocol follows: Coverslips with cells were blocked with 1% bovine serum albumin for 1 h after fixation and permeabilised with 3.7% paraformaldehyde and 0.05% Triton X-100 at room temperature. Samples were incubated with the primary antibody (anti-vimentin, Sigma-Aldrich, Schnelldorf, Germany) for 1 h and subsequently incubated with the secondary antibody tagged with a fluorophore (anti-mouse, Alexa Fluor 546, Thermo Fisher, Waltham, MA, USA) for 1 h. Thereafter, the coverslips were stained with 165 nM Alexa Fluor 488 Phalloidin for 30 min to visualise F-actin filaments, followed by staining with 259 nM DAPI for 15 min to visualise nuclei. Finally, the coverslips were mounted on glass slides and analysed using a 3i Marianas CSU-W1 spinning disc confocal microscope (Intelligent Imaging Innovations GmbH, Göttingen, Germany) with a 63× oil objective, using excitation wavelengths of 488 nm, 561 nm, and 640 nm. The staining agents were chosen so that the interference from the innate autofluorescence of the latex coatings (see Supplementary Material Figure S20) was minimised.

## 3. Results

### 3.1. Properties of the Nanostructured Polymeric Surfaces

The nanostructured polymeric surfaces used in this study were similar to those used to study HDF (human dermal fibroblast) cells in [20] and *S. aureus* in [21]. In addition, protein adsorption has been studied on these polymeric surfaces [23] as well as their use in a cell study platform and a SERS substrate [17,18]. Detailed descriptions of the surface properties, specifically surface roughness and surface chemistry, can be found in [21]. To investigate the influence of surface chemistry and roughness on cell response, these quantities were thoroughly parameterised. In addition, studies of the stiffness of the 50% PS surface have been reported previously [20].

#### 3.1.1. Surface Chemistry of the Polymer Coatings

In previous studies [21], the polymer surfaces have been observed to progress from being moderately hydrophilic at low PS content to becoming (near) hydrophobic (contact angles from 66°–72° to 92°) at high blend ratios (≥70% of PS). This is also observed as a decrease in the polar component of the surface energy towards high-PS% blends (from approx. 10 mJ/m^2^ to near 0 mJ/m^2^). Similarly, there was a slight decrease in the dispersive component and total surface energy for the near-equal blend ratios of the two components of 40–60% PS, where the energy decreased from approximately 42 mJ/m^2^ to 38 mJ/m^2^, with both lower and higher PS% content showing higher surface energy. This change in surface chemistry was likely due to a tendency of the film-forming ABS to cover the PS at low blend ratios. At high PS ratios, there was too little ABS to cover the PS surface, and increasingly large surface areas of PS remained uncovered by ABS.

#### 3.1.2. Surface Properties of the Blend Films

**Figure 1:**
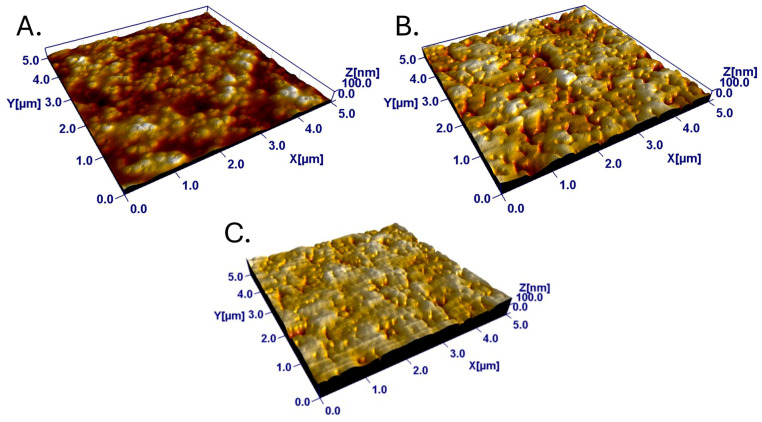
Representative images of the latex surfaces showing the characteristic topographies of (**A**) 30% (left, Z = 65.98 nm), (**B**) 50% (middle, Z = 118.4 nm) and (**C**) 70% (right, Z = 179.9 nm) PS surfaces.

**Figure 2:**
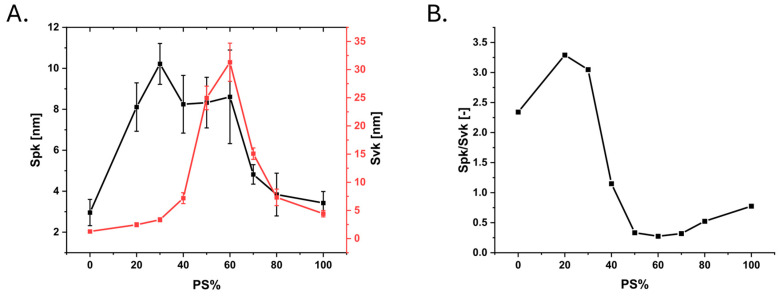
(**A**) The peak and valley contributions compared as variations in reduced peak height (S_pk,_ black) and reduced valley depth (S_vk_, red) with varying PS% in the nanostructured polymer films (left); (**B**) the reduced peak height to reduced valley depth ratio (S_pk_/S_vk_) parameter (right). Error bars indicate the 99% confidence interval.

The surface roughness of the latex surfaces was determined from the AFM images. The roughness of the latex surfaces varied clearly as a function of the mixing ratio of the blend components. The RMS roughness, S_q_, increased from approximately 4 nm for the pure ABS film to a maximum of 15 nm for the 50–60% PS coatings. Similarly, the effective surface area, S_dr_, reached a maximum of 8% at 60% PS. The S_cl37_ varied from about 80 nm to about 110 nm, with a trend roughly opposite to those observed for S_q_ and S_dr_. Representative images of the different surface films are shown in Figure 1. The roughness variations were a result of a rather smooth pure ABS film (low-T_g_ film-forming latex) becoming progressively more textured as the proportion of PS (high-T_g_ non-film-forming latex) increased in the blend film. At around 50% PS, most of the surface area was covered by PS particles (Figure 1B). At even higher PS content, height variations were predominantly caused by valley structures rather than peak structures (see Figure 1 and Figure 2). This can be quantified with the functional parameters S_vk_ (valleys below the core surface) and S_pk_ (peaks above the core surface) and their ratio. The valley dominance described by S_vk_ was smaller than the peak dominance (S_pk_), up until the 40% PS surface. For films with a PS content higher than 40%, the valley depth was greater than the peak height (Figure 2). The domination of peaks over valleys was the strongest for 50–70% PS but diminished towards 100% PS surfaces, where the surfaces consisted of a rather smooth PS film, which was rather weakly valley dominated. For the cell interaction studies, surfaces with a PS content between 20 and 80% were used, since these surfaces had a tangible nanostructure.

The surface chemistry also showed some changes as a function of the mixing ratio of the latex blend. A pure ABS surface was slightly hydrophilic (CA 71.8°) with a surface energy of 43.1 mJ m^−2^, which was negatively polar (11 mJ m^−2^). The overall polarity of the pure PS surfaces was similar in total surface energy (44.6 mJ m^−2^) but nearly non-polar (0.7 mJ m^−2^); this was also seen as a decrease in hydrophilicity (CA approx. 90°). For low-PS% blends, the CA was relatively stable with values between 72 and 77°. A change was seen for 60% PS content, where the hydrophilicity decreased progressively. The surface energy of the bicomponent surfaces was 38–43 mJ m^−2^. The lowest value was observed for the 50% PS blend with a progressively increasing surface energy as more ABS or PS was introduced into the mixture. The negatively polar component of the surface energy was observed to decrease from approx. 80% PS content [21]. The variations in surface energetic properties as a function of mixing ratio of the lattices made it possible to search for correlations between surface energy and cell growth.

### 3.2. Cell Growth on the Latex Surfaces and Reference Materials

The cell viabilities of Hela and HDF cells were measured as end-point measurements relating the number of cells to the number of cells on borosilicate glass references, at the end point after 72 h of incubation. The number of cells was quantified after crystal violet staining (see the Materials and Methods Section). Each surface type was studied in two separate experiments, with four replicate samples each.

**Figure 3:**
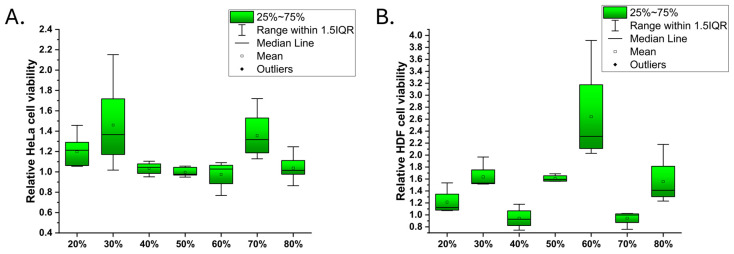
Box charts of the Hela viability (cell numbers at the end point relative to borosilicate glass reference) on the latex samples (**A**); HDF cell data (**B**) on surfaces of the same type [20].

The variations in cell numbers on the different surfaces are presented in Figure 3 (left: HeLa cells; right: HDF cells). The statistical significance of differences between the responses is marked in Supplementary Material Tables S1 and S2. For the Hela cells, the 40–60% and 80% PS samples showed no statistical difference in cell numbers (cutoff *p* = 0.90). However, the 20% PS sample showed an approximately 20% increase, and the 30% PS and 70% PS samples showed an approx. 40% increase in Hela cell numbers compared to the glass reference. The differences were statistically significant for the 40–60% PS on a *p* = 0.95 level or higher. The highest HDF cell viability was measured for the 50–60% PS samples. Moreover, the maximum cell growth of HDF was observed to be higher (approx. 220% on 60% PS) than the maximum observed for the Hela cells (approx. 130% on 30% PS). On the 40% PS surface, the growth appeared most similar for both cells.

#### Cell Morphology and Staining

**Figure 4:**
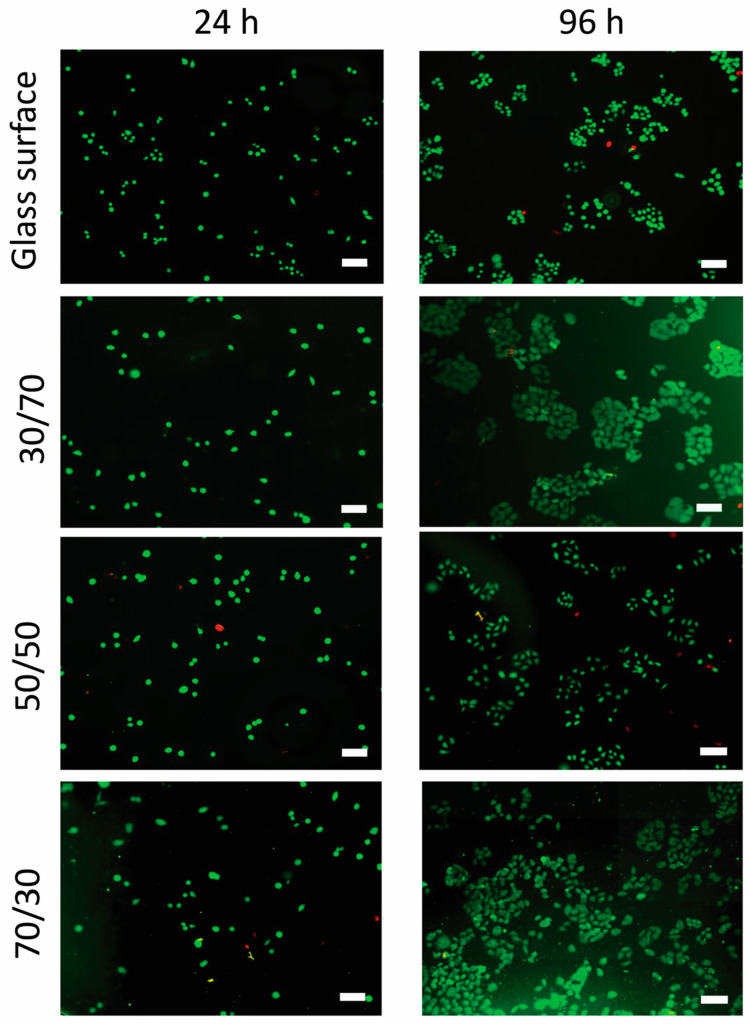
Live (green)/dead (red) staining of the Hela cells grown on a borosilicate glass reference as well as on surfaces made with different PS/ABS blend ratios. Fluorescence imaging was performed at 24 h (left) and 96 h (right). The scale bar is 100 nm.

Although the numbers of cells on the surface were similar at 24 h, images of surfaces with a PS content of 30% and 70% showed clearly higher numbers of live Hela cells than their 50% PS and borosilicate glass counterparts after 96 h of incubation (Figure 4). The 50% PS and glass reference appeared to yield similar cell numbers after this incubation time.

At 24 h, the morphology of the cells grown on the glass reference and 30% PS sample was also different from that observed on the 50% PS and 70% PS samples. On glass and 30% PS samples, the cells had a more spherical shape rather than an irregular shape. A difference between 50% PS and 70% PS was observed after 96 h, when the cells on the glass reference were less spread out than on the other sample surfaces. The Hela cells had a more elongated shape on surfaces of 30% PS and 70% PS, compared to those grown on the borosilicate glass reference surface and 50% PS sample (Figure 4). The elongated morphology and evenly distributed cells correspond well with the viability results that support the high cell viability observed on these surfaces (Figure 3).

**Figure 5:**
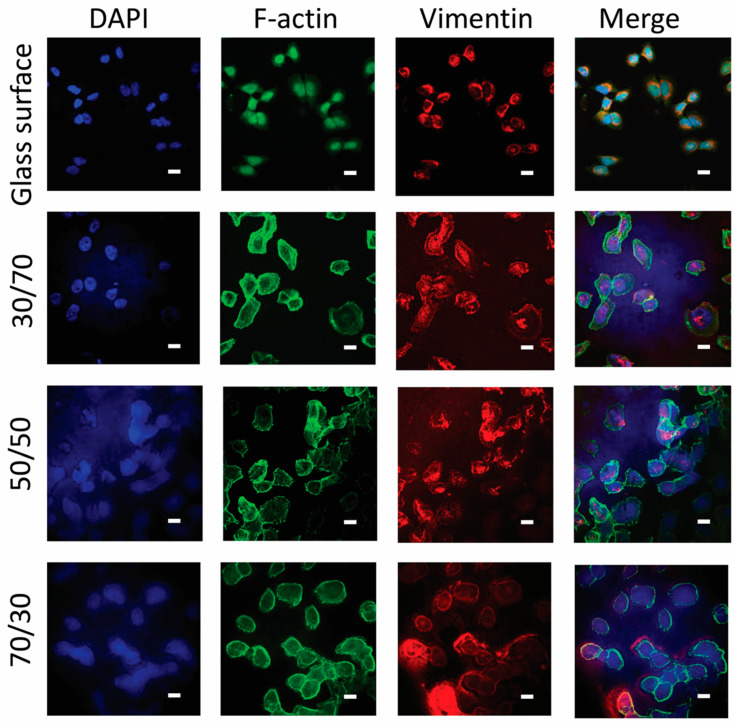
Confocal microscopy images of Hela cells grown on different surfaces: (rows, top to bottom) borosilicate glass reference, as well as latex surfaces at different PS/ABS ratios stained with (columns, left to right) DAPI, F-actin, vimentin, and a merged image of the three staining channels. The shown scale bars are 15 µm.

Confocal microscopy was used to visualise details of the cell morphology and adherence patterns as well as the cellular response to the surfaces (Figure 5). The Hela cells grown on the glass reference had a distinguished localisation of F-actin inside the cells. The 30% PS sample and 70% PS samples have a clear peripheral distribution of the F-actin, whereas the 50% PS samples have more evenly distributed F-actin localisation, and formation of stress fibres can be observed. Z-stack data show approximate thicknesses of 15 µm, indicating a single layer of cells.

The nanostructured latex coatings gave an auto-fluorescent background during fluorescence imaging. F-actin staining showed that Hela cells grown on the borosilicate glass surfaces expressed more nucleus actin compared to the less stiff latex coatings. This could be a response to cellular stress [35].

## 4. Discussion

Analysis of the relative cell viabilities of the Hela cells grown on the nanostructured PS:ABS latex blend surfaces showed statistically significant differences in cell numbers at the 72-h end point. Interestingly, the surface coatings that had been previously observed to yield high cell numbers with HDF cells proved to be relatively poor drivers of Hela cell yield. For Hela cells, the surfaces with 30% and 70% PS content proved to yield the highest cell numbers. In fluorescence imaging, Hela cells grown on the latex surfaces appeared healthy and showed low dead cell counts. At 24 h, the Hela cells were observed to adopt a more spherical shape on 30% and 70% PS surfaces. A spherical shape indicates poor attachment to these two surfaces or that the cells were undergoing mitosis [36,37]. Certain topographies at the nanoscale have also been observed to inhibit cell stretching, thus resulting in cell rounding [38]. On the 30% and 70% PS surfaces, the cells showed a higher elongation, indicating a spreading response of the cells to these surfaces.

Adhesion between the cells and ECM occurs through focal adhesions (Fas), i.e., formed supramolecular complexes. These FAs comprise an integrin signalling layer, a force transduction layer, and an actin regulatory layer. Through this, the cells can sense biochemical and geometrical cues [2]. Clustering of integrins activates focal adhesion kinase (FAK) through autophosphorylation. This causes a reactivation of integrins, which increases their binding strength. Activated FAK also increases the activity of Rac1 and Rap, leading to improved actin polymerisation, dynamic cell adhesion (protrusions) and adhesion maturation [1]. F-actin and vimentin are crucial components of the cytoskeleton, often interacting to provide structural support and maintain the normal morphology of cells. They resist deformation and preserve cellular integrity in response to mechanical stress [39,40,41]. Cytoplasmic F-actin provides structural support, participates in cell motility, and facilitates intracellular transport, while vimentin contributes to cellular integrity and elasticity, promotes cell migration, and aids in organelle positioning [42]. Unlike the network-like structure of cytoplasmic F-actin, nuclear F-actin predominantly forms shorter filaments or clusters within the nucleus, participating in chromatin remodelling and gene expression [43]. In focal adhesions, F-actin is shown to have different functions, including their positioning [44]. Vimentin intermediate filaments have also been associated with the modulation of the structure and function of focal contacts, as well as their size [45]. These observations could be indicative of their roles in the adhesion and surface-sensing of cells to surfaces.

Cell behaviour observed on different material surfaces reflects their response to chemical and mechanical properties [12], as well as their topography, such as size and shape of topographical features [46]. The cellular responses to these different material properties can interplay, due to the complex behaviour of living cells. The higher occurrence of nuclear F-actin observed in the reference sample may be due to the high stiffness and smooth characteristics of the borosilicate glass surface, which impose greater mechanical stress on the cells. Additionally, the smoother surface provides fewer adhesion sites, making it difficult for the Hela cells to form stable adhesion plaques and stress fibres. To counteract this stress, cells may accumulate more F-actin in the nucleus to reinforce the nuclear skeleton, thereby maintaining cellular structural and functional integrity [47]. On the other hand, the latex-coated surfaces with different PS contents exhibited nanoscale roughness and specific chemical properties that promoted cell adhesion and spreading, resulting in more dynamic cytoskeletal reorganisation. The moderate surface energy and hydrophilicity of the 30% and 70% PS samples favoured cell adhesion, resulting in a more pronounced distribution of F-actin at the cell periphery, since cells were more likely to form adhesion patches and stress fibres on these surfaces. In contrast, the 50% PS samples had a more balanced surface energy and roughness, which favoured the formation of more prominent stress fibres due to their uniform and consistent surface properties (Figure 5) [48,49]. Proteins that recognise topographical curvature have been observed to activate downstream signalling components such as N-WASP, Cortactin, and the Arp2/3 complex to form branched actin, suggesting a possible response [46]. The used surfaces have been shown to have a stiffness of 1.6 GPa and 0.13 GPa for PS and ABS, respectively [20]. This can be compared to borosilicate glass, the reference surface, which has a reported stiffness in the 64–89 GPa range [50]. These surfaces can all be considered very rigid compared to the used cells. The elemental composition of the surfaces has been analysed in [22]. On the surfaces, mostly C1s, O1s, Na1s, and S2p were observed (ABS: 76.9%, 15.3%, 5.0%, 2.8%; PS: 96.8%, 2.8%, 0.4%, 0%; 50% PS: 93.6%, 6.4%, 0%, 0%). As has been observed previously, surface properties seemed to influence F-actin localisation and stress fibre formation [20,51,52].

After obtaining the viability data, potential physico-chemical drivers behind the responses of the two cell lines were sought. This was performed by plotting different physical and chemical surface properties against the observed cell viability data.

**Figure 6:**
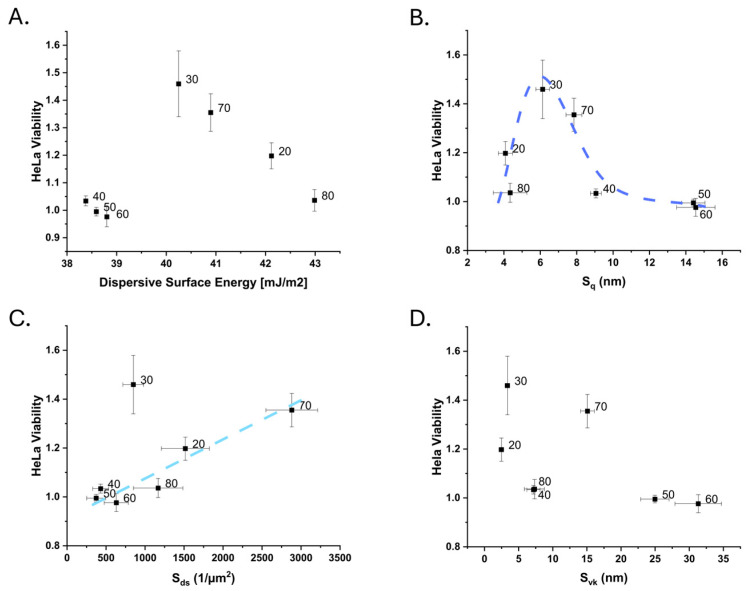
The measured Hela cell viability on the used surfaces plotted against different surface characteristics: (**A**) dispersive surface energy, (**B**) RMS roughness, S_q_, (**C**) density of summits, S_ds_, and (**D**) reduced valley depth S_vk_.

When the viability of the Hela cells was plotted against the dispersive surface energy, the most beneficial surfaces for the Hela cells were those with intermediate dispersive surface energy (Figure 6A). However, two groupings were seen—one for the three roughest samples in terms of S_q_, and one with the less rough samples. That the 80% PS surface had low viability could partially be a result of the lower polar-negative surface energy component (Supplementary Material Figure S19). Using data from HDFs grown on the same surfaces in [20], no correlation was found between the HDF cell data and either the dispersive surface energy or the polar-negative surface energy component (Supplementary Figures S17 and S19).

Similarly to what was observed for HDF cells in [20], the Hela cells showed the highest cell yield for samples with an intermediate S_q_ roughness within the studied range (Figure 6B). The samples with the highest S_q_ roughness had the lowest viability (40–60% PS). The roughness corresponding to the maximum cell yield (approx. 5–8 nm) is lower than what was observed for HDF cells (approx. 11 nm). Other amplitude parameters, like the S_10z_ (Supplementary Material Figure S2), also indicated that a surface with smaller height variations was more likely to give a higher Hela cell yield. For the HDF cells, the opposite observation was made.

The influence of effective surface area (S_dr_, Supplementary Material Figure S3) on Hela cell growth was unclear, whereas it was suggested in [20] to have an optimum value for HDF cells in this range. The autocorrelation length of the surfaces also showed that higher values or a larger lateral roughness was beneficial. The 70% PS sample cannot be described by this, having a rather high cell yield but a short S_cl37_ compared to the studied surfaces. An increasing S_ds_ appeared to drive a rather linear increase in cell yield for all samples but the 30% PS (Figure 6C). These two surfaces, 30% and 70% PS, often appear to stand out in these plots. They share two properties, an intermediate core roughness (S_k_ approx. 12–13 nm) and fractal dimension (S_fd_, approx. 2.13–2.16) (Supplementary Material Figures S6 and S7). Nearly Brownian fractal dimensions (2.5), here measured as S_fd_, have been observed to significantly improve the adhesion of fibroblasts [1] and did not clearly influence the proliferation of Hela cells for these geometries. Plotted against each other, the S_k_, S_ds_ and S_fd_ parameters for these surfaces appeared to have a linear relationship [21].

Utilising functional parameters, the S_vk_ parameter, which describes the prevalence of deep valleys, showed that the high-Hela-cell-yield substrates had a small S_vk_ (Figure 6D). This could be related to the amplitude parameters S_a_ and S_q_, which indicate a preference of the Hela cells for smoother surfaces. As indicated by the S_pk_/S_vk_ parameter, the surfaces with a peak dominance showed a higher cell yield (70% PS being an exception; Supplementary Material Figure S12). This could explain the differences between the observations made here and the previous observations that Hela cells typically prefer rougher surfaces [15,53]. The results here suggest that Hela cells prefer surfaces that have a higher, peak-dominated roughness. Further, this highlights that researchers should use a broad range of parameters to describe their surfaces in cell–surface interaction studies [3,27,54,55].

**Figure 7:**
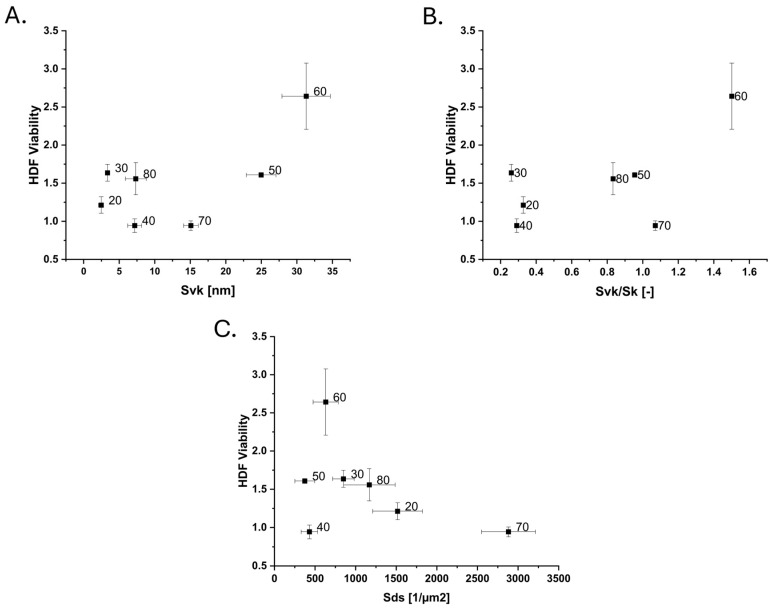
HDF cell viability data from [20] further developed with new roughness parameters: (**A**) reduced valley depth, S_vk_, (**B**) reduced valley depth over core roughness, S_vk_/S_k_, and (**C**) density of summits S_ds_.

Developing the HDF cell yield response investigation performed in [20] with further parameters gave more insight into the driving characteristic of the surfaces. Functional parameters indicated that HDFs benefit from surfaces with valley-driven roughness (Figure 7B), contrary to what was observed for Hela cells. This is also indicated by the viability–S_pk_/S_vk_ plot (Supplementary Material Figure S12) where particularly low values, i.e., valley-dominated samples, more likely caused increased viability compared to moderate and high values. While showing large variations for these surfaces, the S_pk_ (Supplementary Material Figure S8) and S_vk_ (Figure 7A), however, show that surfaces with both peak and valley domination may benefit HDF proliferation. Unlike what was observed for Hela cells, the S_ds_ appeared to proportionally decrease the HDF viability (Figure 7C). While an S_fd_ closer to Brownian (2.5) has been observed to be preferential for HDF cells through its possible activation of FAK signalling [1], such an observation could not be made from these data. We have previously observed that S_ds_ and S_fd_ seem to describe similar surface features for these surfaces [21]. As S_ds_ describes the number of local maxima (i.e., peaks) separated by lower regions, a de facto correlation between S_ds_ and S_fd_ could thus indicate that the S_fd_ characteristic has more relevance for peak-driven topographies. However, this would need to be investigated further.

Previously, the surface energy influence on cellular attachment has been investigated as a polar/dispersive ratio by, e.g., [56], with the observation that a higher polar/dispersive surface energy ratio was better for African green monkey kidney fibroblast COS7 and human osteosarcoma MG63 growth. Similarly, investigations of the polar surface free energy component have shown it to be an important factor supporting cytoactivity [57,58]. Satriano et al. observed that the ratio of different surface free energy components critically altered the conditioning proteinaceous film forming on a surface in a protein solution [59]. Indeed, differences in polarity are commonly associated with differences in the accumulation, alignment, and activity of the surface proteins of the cells and the ECM [57,58]. Similar approaches have also been used for bacterial cells, for which the interaction of bacteria with surfaces has been observed to correlate with the ratio of the dispersive and the electron donor surface energy component of the substrate in extended DLVO in silica studies [60,61,62]. Several accounts on the influence of surface free energy alone have been performed, indicating that a higher surface free energy benefits adhesion and spreading, while lower surface free energies can inhibit cell behaviour [49]. In silica studies of the influence of roughness on the interaction energy of particles showed that the interaction energy is more influenced by roughness when the particles are smaller than the topographical features, and vice versa [6].

**Figure 8:**
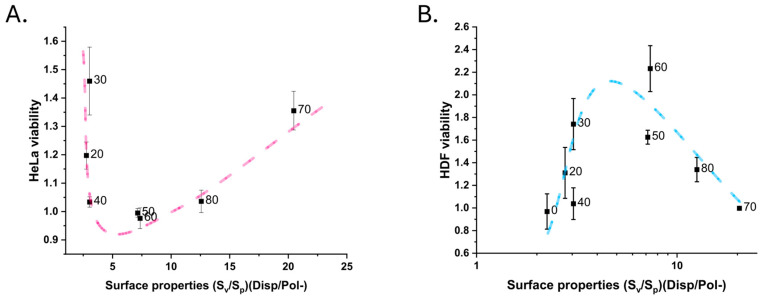
Viability versus surface property plots for (**A**) Hela cells and (**B**) HDF cells. The combined surface properties account for peak and valley properties with S_v_ and S_p_, as well as the surface energy with the dispersive surface energy (Disp) and polar-negative (Pol-) surface energy component from the acid–base theory. Trend lines illustrate the authors’ interpretation.

Based on these results, we then searched for a rudimentary combination of surface characteristic parameters that could account for how nanotopography and surface energy affect the viability of both Hela and HDF cells: $\frac{S_{v}}{S_{p}}\cdot \frac{\sigma_{Disp}}{\sigma_{Pol}-}$ (Figure 8).

The developed description tries to take into account both the roughness and the surface energy of the surface. Specifically, this combination accounts for the relative abundances of valleys and peaks $\left(\frac{Sv}{Sp}\right)$, as well as the amplitude of said features (above or below the core roughness), as well as the ratio of Lifshitz–van-der-Waal and the electron donor component of the surface free energy $\left(\frac{\sigma_{Disp}}{\sigma_{Pol-}}\right)$, assuming the surfaces are at least slightly polar. Low values indicate high peaks and/or surfaces with a high electron donor component, and vice versa. The dispersive surface energy is always higher, at 38–44 mJ/m^2^, than the relatively stable electron donor component, which is 10–11 mJ/m^2^. As such, changes in the surface properties are dominated by the roughness descriptor, the peak-height-to-valley-depth ratio. Whether this analysis is also suitable for other combinations of surfaces and cell lines would need to be affirmed with separate studies. Nonetheless, it highlights the opposite responses of Hela and HDF cells to these nanostructured surfaces, likely owing to their differences in the tissue microenvironment.

According to this interpretation, the Hela cells benefited from a nanotopography with clearly dominating peaks or valleys, while the HDF cells preferred surfaces where neither peaks nor valleys clearly dominated when the influence of surface free energy was accounted for. This could explain the ambiguity in the HDF response when studying cell–surface interactions using only amplitude roughness. Similarly, a dominating dispersive surface energy or electron donor component of the surface free energy could be beneficial for the Hela cells, while a balanced surface energy ratio appeared to have been preferred by the HDF cells. To solidify these observations, HDF and Hela interactions with surfaces with a wider range of surface topographies and energies would need to be investigated. In such cases, computer tools such as design of experiments would be very useful. Roughness parameters accounting for the density/spacing of asperities (e.g., peak density, S_pd_, and autocorrelation length, S_al_) should also be considered in a truly holistic approach, even though they did not appear to influence the response of the cell lines in this study, as they can describe features that may influence the initial attachment of cells. Correlating changes in the surface proteome of the studied cells with different surface properties would also add a crucial level of detail to the understanding of cell–surface interactions, even on a molecular level.

## 5. Conclusions

In this study, the viability of Hela cells grown over a period of 72 h on a set of nanostructured bicomponent latex surfaces was studied, and the responses to surface features were compared to those of HDF cells studied on similar surfaces [20]. The observed end-point viabilities relative to those on borosilicate glass coverslips were analysed from the perspective of surface chemistry and several roughness parameters. The HDF study was also expanded using some additional roughness parameters found relevant for Hela cells and in biofilm studies [21].

By adjusting the mixing ratio of the two components, PS and ABS, the roughness of the resultant surfaces could be tuned in the nanometre range (S_q_ approx. 4–15 nm). Additionally, the roughness of the surfaces shifted from being peak-dominated to being valley-dominated. The dispersive component of the surface energy of the surfaces varied between 38 and 43 mJ/m^2^. The polar-negative component of the surface energy was approx. 10 mJ/m^2^ for most surfaces, while it was lower for the 70% PS and 80% PS surfaces—approx. 8 mJ/m^2^ and 6 mJ/m^2^, respectively. The variations in the surface properties made the surfaces interesting candidates for studying cell–surface interactions. That said, being able to adjust the surface chemistry and topography independently would have made decoupling the parameters easier.

It was observed that Hela cells respond to nanoscale differences, surface roughness, and surface chemistry, like other cell lines [1], with changes in attachment, proliferation, and also ECM component expression. The role of actin, e.g., has actively been investigated in carcinogenesis [37,38,39,40]. This highlights the need for in vitro conditions that are chosen carefully to avoid misleading results.

Of the surface chemical parameters, the dispersive surface energy component appeared to influence Hela viability. A lower dispersive surface energy tended to increase the cell proliferation yield. The effect of roughness also appeared very small in comparison to the dispersive surface energy, but a rougher surface (S_q_ and S_dr_) likely decreases the cell yield contrary to what has been previously reported [8]. Other amplitude parameters (e.g., S_10z_ and S_p_) showed a similar trend. A peak- or valley-dominated character of surfaces and its effect on viability were seen using the functional parameters S_pk_, S_vk_ and S_pk_/S_vk_. This could possibly be due to the valley-dominated features of the studied surfaces, which had the largest height variations of the length scale of the roughness studied—the S_q_ was observed to be merely 16 nm at its highest. When accounted for together, these results propose that rather than roughness amplitude, the cell-yield-driving surface characteristic is the peak or valley dominance of a surface.

Investigating the HDF response with these parameters allowed for several interesting observations beyond what was previously disseminated in [20]. First, the HDF appeared to be little affected by surface chemistry. But on the other hand, a higher cell yield was caused by surfaces with either clear peak or valley dominance, and of these two, a more valley-driven roughness was preferred. The previous observations showing an optimum cell yield at average S_q_ values were thus likely a result of the surfaces not having a clearly peak- or valley-driven topography.

These observations highlight the necessity of using multiple surface parameters to properly examine the response of cells to surface properties, and how using these parameters can provide more meaningful conclusions if they are chosen with care [63]. In this case, the use of functional parameters was critical to develop the understanding of cellular growth responses to nanostructured surfaces. They also highlight how different cell lines can respond dramatically differently to different surface characteristics.

This perspective on surface properties might explain a difference between the observations presented here and previous, more general, observations that increasing nanoscale surface roughness is beneficial for cancerous cell types, including Hela cells [1,15,53]. Ultimately, these observations could aid the development of new surface materials for tailored biological applications.

This study underscores the importance of tailored material design in biomedical applications, where surface properties can be fine-tuned to optimise cell behaviour, potentially leading to improved outcomes in regenerative tissue engineering, treatment of wound healing, cancer research, and the development of biomaterials for other types of therapeutic use.

## Data Availability

Dataset available on request from the authors.

## Supplementary Materials

The following supporting information can be downloaded at https://www.mdpi.com/article/10.3390/nano15100716/s1: Supplementary Material Table S1: Paired *p*-values for the HeLa cell viabilities measured, sorted according to sample substrate. Supplementary Material Table S2: Significant differences between the HeLa cell viabilities observed on different samples. Supplementary Material Table S3: Raw data of Hela viabilities. Supplementary Material Figure S1: The relative Hela cell yield vs. the S_q_ parameter. Supplementary Material Figure S2: The relative Hela cell yield (left) and relative HDF cell yield vs. the S_10z_ parameter. Supplementary Material Figure S3: The relative Hela cell yield vs. the S_dr_ parameter. Supplementary Material Figure S4: The relative Hela cell yield vs. the S_cl37_ parameter. Supplementary Material Figure S5: The relative Hela cell yield (left) and relative HDF cell yield vs. the S_ds_ parameter. Supplementary Material Figure S6: The relative Hela cell yield (left) and relative HDF cell yield vs. the S_fd_ parameter. Supplementary Material Figure S7: The relative Hela cell yield (left) and relative HDF cell yield vs. the S_k_ parameter. Supplementary Material Figure S8: The relative Hela cell yield (left) and relative HDF cell yield vs. the S_pk_ parameter. Supplementary Material Figure S9: The relative Hela cell yield (left) and relative HDF cell yield vs. the S_vk_ parameter. Supplementary Material Figure S10: The relative Hela cell yield (left) and relative HDF cell yield vs. the S_pk_/S_k_ parameter. Supplementary Material Figure S11: The relative Hela cell yield (left) and relative HDF cell yield vs. the S_vk_/S_k_ parameter. Supplementary Material Figure S12: The relative Hela cell yield (left) and relative HDF cell yield vs. the S_pk_/S_vk_ parameter. Supplementary Material Figure S13: The relative Hela cell yield (left) and relative HDF cell yield vs. the S_bi_ parameter. Supplementary Material Figure S14: The relative Hela cell yield (left) and relative HDF cell yield vs. the S_vi_ parameter. Supplementary Material Figure S15: The relative Hela cell yield (left) and relative HDF cell yield vs. the Z_0.05_ parameter. Supplementary Material Figure S16: The relative Hela cell yield (left) and relative HDF cell yield vs. the water contact angle. Supplementary Material Figure S17: The relative Hela cell yield (left) and relative HDF cell yield vs. the dispersive surface energy component. Supplementary Material Figure S18: Linear fits for the two groupings found in the plot of the viability against the dispersive energy of the surfaces: right, the rougher surfaces (40%, 50%, and 60% PS) and left, the rest. Supplementary Material Figure S19: The relative Hela cell yield (left) and relative HDF cell yield vs. the polar-negative surface energy component. Supplementary Material Figure S20: Fluorescence spectra of 50% PS latex showing its intrinsic autofluorescence. Supplementary Figure S21: Illustration of the material ratio curve of a 5 µm × 5 µm images of 50% PS latex with marked Spk and Svk definitions. Supplementary Figure S22: Aspect ratio (length/width) of the Live Hela cells on different surfaces after 96 h incubation.

## Abbreviations

The following abbreviations are used in this manuscript:

ABS	Acrylonitrile butadiene styrene
AFM	Atomic force microscopy
CA	Contact angle
CAF	Cancer-associated fibroblast
CV	Crystal violet
DAPI	4’,6-diamidino-2-phenylindole
DIM	Diiodo methane
DMEM	Dulbecco’s modified Eagle medium
EG	Ethylene glycol
HDF	Human dermal fibroblast
Hela	Human cervical cancer cells
IR	Infrared
PS	Polystyrene
S_a_	Arithmetic average roughness
S_cl37_	Correlation length to 37%
S_dr_	Effective surface area
SDS	Sodium dodecyl sulphate
S_fd_	Fractal dimension
S_k_	Core roughness
S_ku_	Kurtosis
S_q_	Root mean square height variation roughness
S_pd_	Peak density
S_pk_	Reduced peak height
S_sk_	Skewness
S_vk_	Reduced valley depth
T_g_	Glass transition temperature

## References

[1] Li Y., Yin X., Changsheng L. (2017). The Horizon of Materiobiology: A Perspective on Material-Guided Cell Behaviors and Tissue Engineering. Chem. Rev..

[2] Rostam H.M., Singh S., Vrana N.E., Alexander M.R., Ghaemmaghami A.M. (2015). Impact of Surface Chemistry and Topography on the Function of Antigen Presenting Cells. Biomater. Sci..

[3] Crawford R.J., Webb H.K., Truong V.K., Hasan J., Ivanova E.P. (2012). Surface Topographical Factors Influencing Bacterial Attachment. Adv. Colloid Interface Sci..

[4] Webb H.K., Truong V.K., Hasan J., Fluke C., Crawford R.J., Ivanova E.P. (2012). Roughness Parameters for Standard Description of Surface Nanoarchitecture. Scanning.

[5] Whitehouse D.J. (2010). Handbook of Surface and Nanometrology.

[6] Ferreira F.V., Sassi L.M., de Souza Camargo S. (2023). A Computer Simulation Study of Extended DLVO Interactions between Calcite Nanoparticles and Real Rough Surfaces. Surf. Coat. Technol..

[7] Wang M., Zhao J., Zhang L., Wei F., Lian Y., Wu Y., Gong Z., Zhang S., Zhou J., Cao K. (2017). Role of Tumor Microenvironment in Tumorigenesis. J. Cancer.

[8] Morán M.C., Ruano G., Cirisano F., Ferrari M. (2019). Mammalian Cell Viability on Hydrophobic and Superhydrophobic Fabrics. Mater. Sci. Eng. C.

[9] Rahbari R., Sheahan T., Modes V., Collier P., Macfarlane C., Badge R.M. (2009). A Novel L1 Retrotransposon Marker for HeLa Cell Line Identification. BioTechniques.

[10] Lin J., Liu X., Ding D. (2015). Evidence for Epithelial-Mesenchymal Transition in Cancer Stem-like Cells Derived from Carcinoma Cell Lines of the Cervix Uteri. Int. J. Clin. Exp. Pathol..

[11] Best J.P., Javed S., Richardson J.J., Cho K.L., Kamphuis M.M.J., Caruso F. (2013). Stiffness-Mediated Adhesion of Cervical Cancer Cells to Soft Hydrogel Films. Soft Matter.

[12] Majhy B., Priyadarshini P., Sen A.K. (2021). Effect of Surface Energy and Roughness on Cell Adhesion and Growth—Facile Surface Modification for Enhanced Cell Culture. RSC Adv..

[13] Schneider A., Francius G., Obeid R., Schwinté P., Hemmerlé J., Frisch B., Schaaf P., Voegel J.-C., Senger B., Picart C. (2006). Polyelectrolyte Multilayers with a Tunable Young’s Modulus: Influence of Film Stiffness on Cell Adhesion. Langmuir ACS J. Surf. Colloids.

[14] Schrade A., Mailänder V., Ritz S., Landfester K., Ziener U. (2012). Surface Roughness and Charge Influence the Uptake of Nanoparticles: Fluorescently Labeled Pickering-Type versus Surfactant-Stabilized Nanoparticles. Macromol. Biosci..

[15] Han J., Menon N.V., Kang Y., Tee S.-Y. (2015). An in Vitro Study on the Collective Tumor Cell Migration on Nanoroughened Poly(Dimethylsiloxane) Surfaces. J. Mater. Chem. B.

[16] Ishihara S., Haga H. (2022). Matrix Stiffness Contributes to Cancer Progression by Regulating Transcription Factors. Cancers.

[17] Rosqvist E., Niemelä E., Frisk J., Öblom H., Koppolu R., Abdelkader H., Soto Véliz D., Mennillo M., Venu A.P., Ihalainen P. (2020). A Low-Cost Paper-Based Platform for Fast and Reliable Screening of Cellular Interactions with Materials. J. Mater. Chem. B.

[18] Rosqvist E., Böcker U., Gulin-Sarfraz T., Afseth N.K., Tolvanen S., Peltonen J., Sarfraz J. (2023). Low-Cost, Mass-Producible Nanostructured Surface on Flexible Substrate with Ultra-Thin Gold or Silver Film for SERS Applications. Nano-Struct. Nano-Objects.

[19] Järnström J., Ihalainen P., Backfolk K., Peltonen J. (2008). Roughness of Pigment Coatings and Its Influence on Gloss. Appl. Surf. Sci..

[20] Rosqvist E., Niemelä E., Venu A.P., Kummala R., Ihalainen P., Toivakka M., Eriksson J.E., Peltonen J. (2019). Human Dermal Fibroblast Proliferation Controlled by Surface Roughness of Two-Component Nanostructured Latex Polymer Coatings. Colloids Surf. B Biointerfaces.

[21] San-Martin-Galindo P., Rosqvist E., Tolvanen S., Miettinen I., Savijoki K., Nyman T.A., Fallarero A., Peltonen J. (2021). Modulation of Virulence Factors of *Staphylococcus aureus* by Nanostructured Surfaces. Mater. Des..

[22] Juvonen H., Määttänen A., Ihalainen P., Viitala T., Sarfraz J., Peltonen J. (2014). Enhanced Protein Adsorption and Patterning on Nanostructured Latex-Coated Paper. Colloids Surf. B Biointerfaces.

[23] Juvonen H., Oja T., Määttänen A., Sarfraz J., Rosqvist E., Riihimäki T.A., Toivakka M., Kulomaa M., Vuorela P., Fallarero A. (2015). Protein and Bacterial Interactions with Nanostructured Polymer Coatings. Colloids Surf. B Biointerfaces.

[24] Spiro J.G., Farinha J.P.S., Winnik M.A. (2003). Thermodynamics and Morphology of Latex Blend Films. Macromolecules.

[25] Peltonen J., Järn M., Areva S., Linden M., Rosenholm J.B. (2004). Topographical Parameters for Specifying a Three-Dimensional Surface. Langmuir.

[26] (2011). Geometrical product specifications (GPS)—Filtration, Part 21: Linear Profile Filters: Gaussian Filters.

[27] Whitehouse D., Whitehouse D. (2002). 3—Profile and Areal (3D) Parameter Characterization. Surfaces and Their Measurement.

[28] Żak K., Grzesik W. (2017). Metrological Aspects of Surface Topographies Produced by Different Machining Operations Regarding Their Potential Functionality. Metrol. Meas. Syst..

[29] Makkonen L. (2018). Faulty Intuitions of Wetting. Int. J. Wettability Sci. Technol..

[30] Makkonen L. (2016). Young’s Equation Revisited. J. Phys. Condens. Matter.

[31] Shardt N., Elliott J.A.W. (2020). Gibbsian Thermodynamics of Wenzel Wetting (Was Wenzel Wrong? Revisited). Langmuir.

[32] Van Oss C.J., Chaudhury M.K., Good R.J. (1988). Interfacial Lifshitz-van Der Waals and Polar Interactions in Macroscopic Systems. Chem. Rev..

[33] Oss C.J.V., Good R.J., Chaudhury M.K. (1986). The Role of van Der Waals Forces and Hydrogen Bonds in “Hydrophobic Interactions” between Biopolymers and Low Energy Surfaces. J. Colloid Interface Sci..

[34] Schindelin J., Arganda-Carreras I., Frise E., Kaynig V., Longair M., Pietzsch T., Preibisch S., Rueden C., Saalfeld S., Schmid B. (2012). Fiji: An Open-Source Platform for Biological-Image Analysis. Nat. Methods.

[35] Kelpsch D.J., Tootle T.L. (2018). Nuclear Actin: From Discovery to Function. Anat. Rec..

[36] Girshovitz P., Shaked N.T. (2012). Generalized Cell Morphological Parameters Based on Interferometric Phase Microscopy and Their Application to Cell Life Cycle Characterization. Biomed. Opt. Express.

[37] Cadart C., Zlotek-Zlotkiewicz E., Berre M.L., Piel M., Matthews H.K. (2014). Exploring the Function of Cell Shape and Size during Mitosis. Dev. Cell.

[38] Ferrari M., Cirisano F., Morán M.C. (2019). Mammalian Cell Behavior on Hydrophobic Substrates: Influence of Surface Properties. Colloids Interfaces.

[39] Tang D., Zhang Y., Mei J., Zhao J., Miao C., Jiu Y. (2023). Interactive Mechanisms between Caveolin-1 and Actin Filaments or Vimentin Intermediate Filaments Instruct Cell Mechanosensing and Migration. J. Mol. Cell Biol..

[40] Smoler M., Coceano G., Testa I., Bruno L., Levi V. (2020). Apparent Stiffness of Vimentin Intermediate Filaments in Living Cells and Its Relation with Other Cytoskeletal Polymers. Biochim. Biophys. Acta (BBA)—Mol. Cell Res..

[41] Jensen M.H., Morris E.J., Goldman R.D., Weitz D.A. (2014). Emergent Properties of Composite Semiflexible Biopolymer Networks. BioArchitecture.

[42] Dominguez R., Holmes K.C. (2011). *Actin* Structure and Function. Annu. Rev. Biophys..

[43] Kloc M., Chanana P., Vaughn N., Uosef A., Kubiak J.Z., Ghobrial R.M. (2021). New Insights into Cellular Functions of Nuclear Actin. Biology.

[44] Kumari R., Ven K., Chastney M., Kokate S.B., Peränen J., Aaron J., Kogan K., Almeida-Souza L., Kremneva E., Poincloux R. (2024). Focal Adhesions Contain Three Specialized *Actin* Nanoscale Layers. Nat. Commun..

[45] Tsuruta D., Jones J.C.R. (2003). The Vimentin Cytoskeleton Regulates Focal Contact Size and Adhesion of Endothelial Cells Subjected to Shear Stress. J. Cell Sci..

[46] Lou H.-Y., Zhao W., Li X., Duan L., Powers A., Akamatsu M., Santoro F., McGuire A.F., Cui Y., Drubin D.G. (2019). Membrane Curvature Underlies Actin Reorganization in Response to Nanoscale Surface Topography. Proc. Natl. Acad. Sci. USA.

[47] Record J., Saeed M.B., Venit T., Percipalle P., Westerberg L.S. (2021). Journey to the Center of the Cell: Cytoplasmic and Nuclear Actin in Immune Cell Functions. Front. Cell Dev. Biol..

[48] Stricker J., Falzone T., Gardel M.L. (2010). Mechanics of the F-Actin Cytoskeleton. J. Biomech..

[49] Cai S., Wu C., Yang W., Liang W., Yu H., Liu L. (2020). Recent Advance in Surface Modification for Regulating Cell Adhesion and Behaviors. Nanotechnol. Rev..

[50] (2007). Mechanical Strength of Glass. Glass.

[51] Walcott S., Sun S.X. (2010). A Mechanical Model of Actin Stress Fiber Formation and Substrate Elasticity Sensing in Adherent Cells. Proc. Natl. Acad. Sci. USA.

[52] Zhou K., Li Y., Zhang L., Jin L., Yuan F., Tan J., Yuan G., Pei J. (2021). Nano-Micrometer Surface Roughness Gradients Reveal Topographical Influences on Differentiating Responses of Vascular Cells on Biodegradable Magnesium. Bioact. Mater..

[53] Gentile F., Tirinato L., Battista E., Causa F., Liberale C., Fabrizio E.M.d., Decuzzi P. (2010). Cells Preferentially Grow on Rough Substrates. Biomaterials.

[54] Bhushan B. (2000). Surface Roughness Analysis and Measurement Techniques. Modern Tribology Handbook.

[55] Hansson K.N., Hansson S. (2011). Skewness and Kurtosis: Important Parameters in the Characterization of Dental Implant Surface Roughness—A Computer Simulation. ISRN Mater. Sci..

[56] Ong S.-E., Zhang S., Du H., Wang Y., Ma L.-L. (2008). In-Vitro Cellular Behavior on Amorphous Carbon Containing Silicon. Thin Solid Films.

[57] Nakamura M., Hori N., Ando H., Namba S., Toyama T., Nishimiya N., Yamashita K. (2016). Surface Free Energy Predominates in Cell Adhesion to Hydroxyapatite through Wettability. Mater. Sci. Eng. C.

[58] Kubies D., Himmlová L., Riedel T., Mázl Chánová E., Balík K., Douděrová M., Bártová J., Pešáková V. (2010). The Interaction of Osteoblasts With Bone-Implant Materials: 1. The Effect of Physicochemical Surface Properties of Implant Materials. Physiol. Res. Acad. Sci. Bohemoslov..

[59] Satriano C., Carnazza S., Guglielmino S., Marletta G. (2003). Surface Free Energy and Cell Attachment onto Ion-Beam Irradiated Polymer Surfaces. Nucl. Instrum. Methods Phys. Res. Sect. B Beam Interact. Mater. At..

[60] Boulange-Petermann L., Baroux B., Bellon-Fontaine M.-N. (1993). The Influence of Metallic Surface Wettability on Bacterial Adhesion. J. Adhes. Sci. Technol..

[61] Liu C., Zhao Q. (2011). The CQ Ratio of Surface Energy Components Influences Adhesion and Removal of Fouling Bacteria. Biofouling.

[62] Liu C., Zhao Q. (2011). Influence of Surface-Energy Components of Ni–P–TiO_2_–PTFE Nanocomposite Coatings on Bacterial Adhesion. Langmuir.

[63] Whitehouse D.J. (2001). Fractal or Fiction. Wear.

