# Supplementary material for: Proliferation of Human Cervical Cancer Cells Responds to Surface Properties of Bicomponent Polymer Coatings
Source: Nanomaterials (Basel). 2025 May 9;15(10):716. doi: 10.3390/nano15100716 (PMC12114405; doi:10.3390/nano15100716)
Supplement: Supplementary file 1 [file nanomaterials-15-00716-s001.zip › nanomaterials-3602472-supplementary.pdf]

Supplementary Information

Table S1 - p-values for the HeLa cell viabilities measured, sorted according to sample substrate.

p-values	20	30	40	50	60	70	80
**20**		0.1141	0.0114	0.0075	0.0062	0.1275	0.0429
**30**	0.1141		0.0078	0.0129	0.0043	0.5337	0.0154
**40**	0.0114	0.0078		0.1941	0.2340	0.0010	0.9593
**50**	0.0075	0.0129	0.1941		0.7210	0.0017	0.4430
**60**	0.0062	0.0043	0.2340	0.7210		0.0007	0.3464
**70**	0.1275	0.5337	0.0010	0.0017	0.0007		0.0043
**80**	0.0429	0.0154	0.9593	0.4430	0.3464	0.0043	

Table S2 - Significant differences between the HeLa cell viabilities observed on different samples according to a star (*) rating. One star (*) indicates a statistically significant difference of at least 90%, two stars (**) indicates a statistically significant difference of at least 95%, and three stars (***) indicates a statistically significant difference of at least 99%.

	20	30	40	50	60	70	80
**20**		-	**	***	***	-	**
**30**	-		***	**	***	-	**
**40**	**	***		-	-	***	-
**50**	***	**	-		-	***	-
**60**	***	***	-	-		***	-
**70**	-	-	***	***	***		***
**80**	**	**	-	-	-	***	

Table S3 - Raw data for HeLa cells

PS	20	30	40	50	60	70	80
	1.218257	2.151867	1.087137	0.948548	1.056432	1.186722	1.053112
	1.061411	1.498755	0.960996	0.970954	1.090456	1.128631	1.111203
	1.054772	1.66805	1.007469	1.056432	1.042324	1.226556	0.975934
	1.212448	1.766805	1.06888		0.767635	1.378423	1.246473
		1.01733	1.066511	0.96815	0.911944		
	1.088993	1.232319	1.022951	0.980796	0.855738	1.528806	0.864169
	1.28993	1.183138	0.951288	1.044028	1.072131	1.316628	0.987822
	1.457143	1.157845	1.10445		1.013115	1.719906	1.01452
**Ave**	**1.197565**	**1.459514**	**1.03371**	**0.994818**	**0.976222**	**1.355096**	**1.036176**
**SEM**	**0.047674**	**0.1198**	**0.017895**	**0.015303**	**0.036623**	**0.068328**	**0.039225**

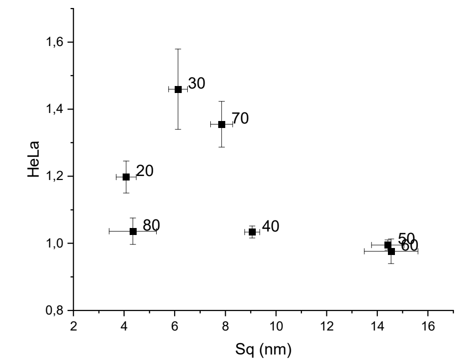

See: “Human dermal fibroblast proliferation controlled by surface roughness of two-component nanostructured latex polymer coatings”

Supplementary Figure S1—The relative Hela cell yield vs the S_q parameter.

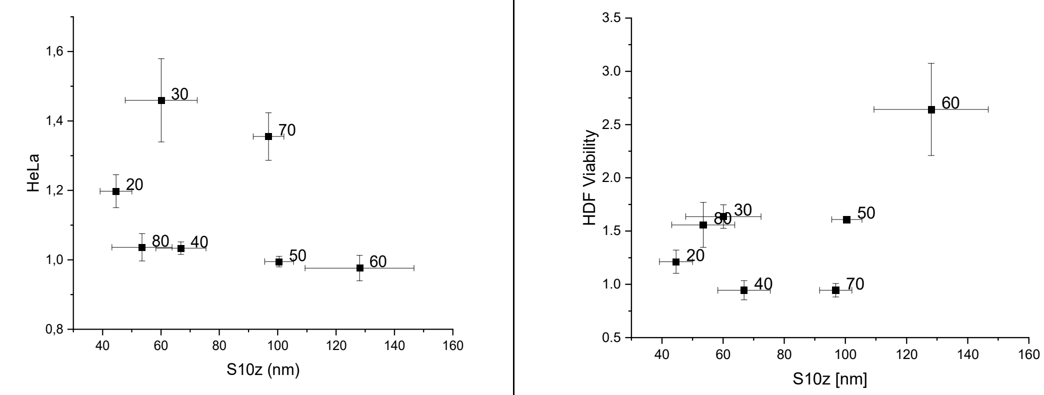

Supplementary Figure S2 — The relative Hela cell yield (left) respectively relative HDF cell yield vs the S_{10z} parameter.

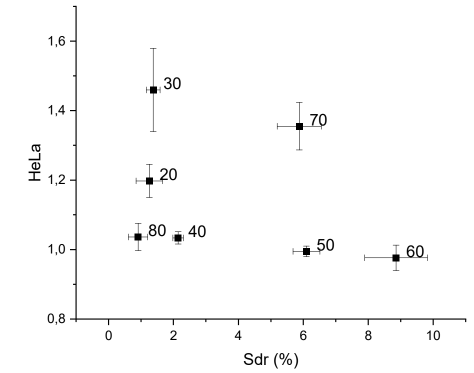

See: “Human dermal fibroblast proliferation controlled by surface roughness of two-component nanostructured latex polymer coatings”

Supplementary Figure S3— The relative Hela cell yield vs the S_{dr} parameter.

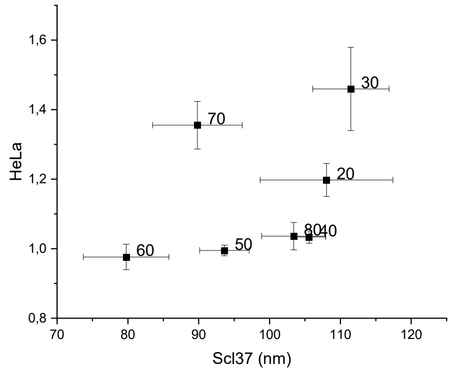

See: “Human dermal fibroblast proliferation controlled by surface roughness of two-component nanostructured latex polymer coatings”

Supplementary Figure S4— The relative Hela cell yield vs the S_{cl37} parameter.

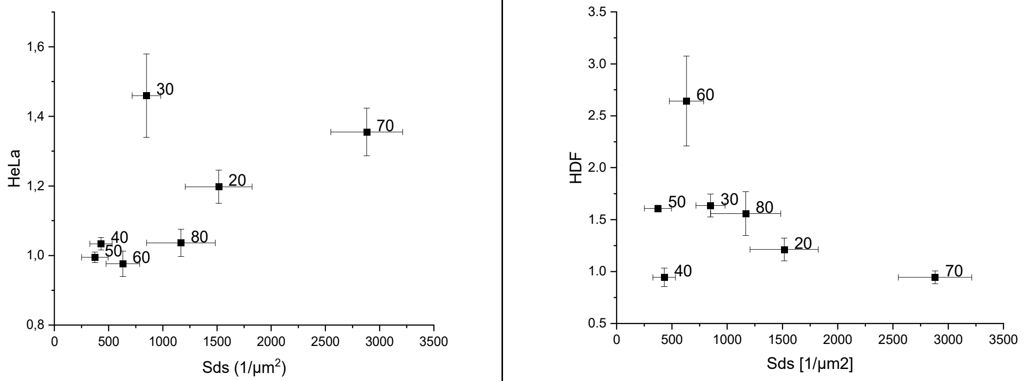

Supplementary Figure S5— The relative Hela cell yield (left) respectively relative HDF cell yield vs the S_{ds} parameter.

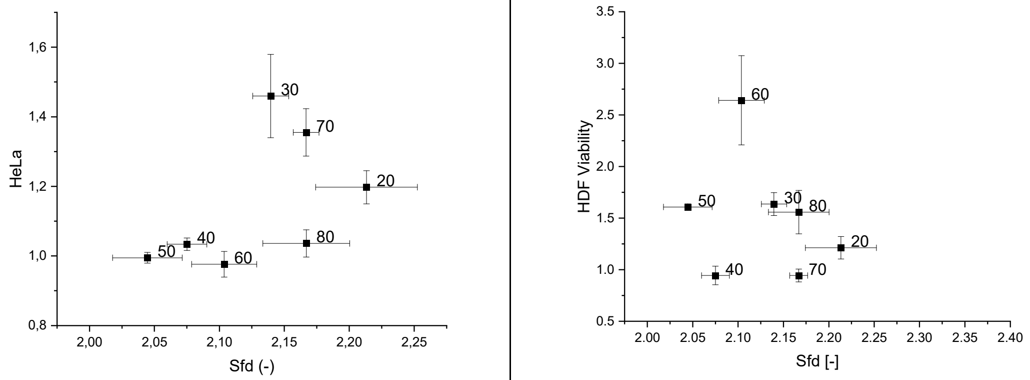

Supplementary Figure S6— The relative Hela cell yield (left) respectively relative HDF cell yield vs the S_{fd} parameter.

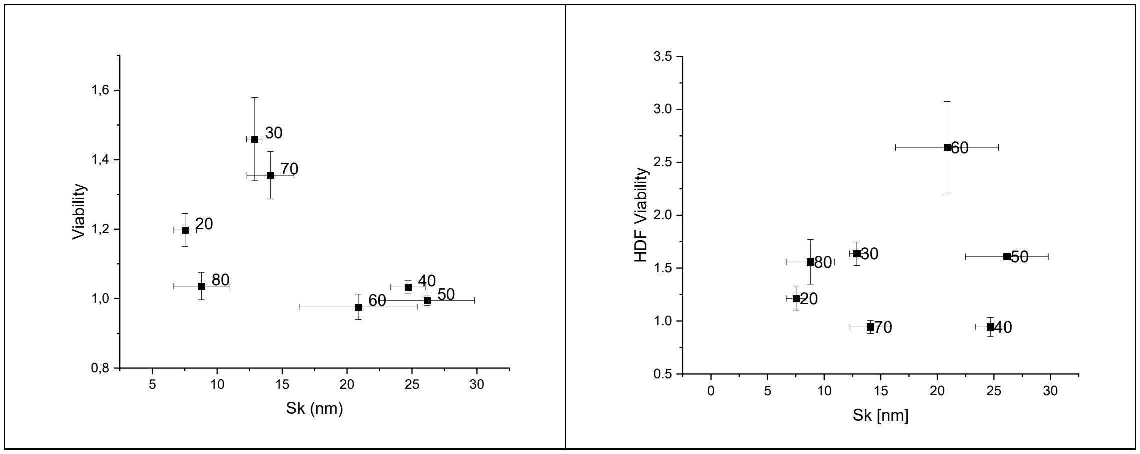

Supplementary Figure S7— The relative Hela cell yield (left) respectively relative HDF cell yield vs the S_k parameter.

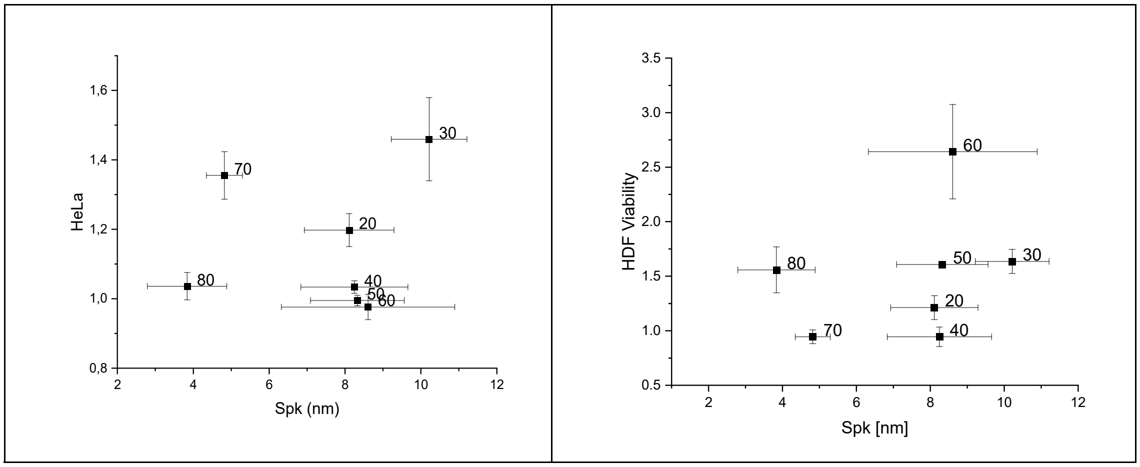

Supplementary Figure S8— The relative Hela cell yield (left) respectively relative HDF cell yield vs the S_{pk} parameter.

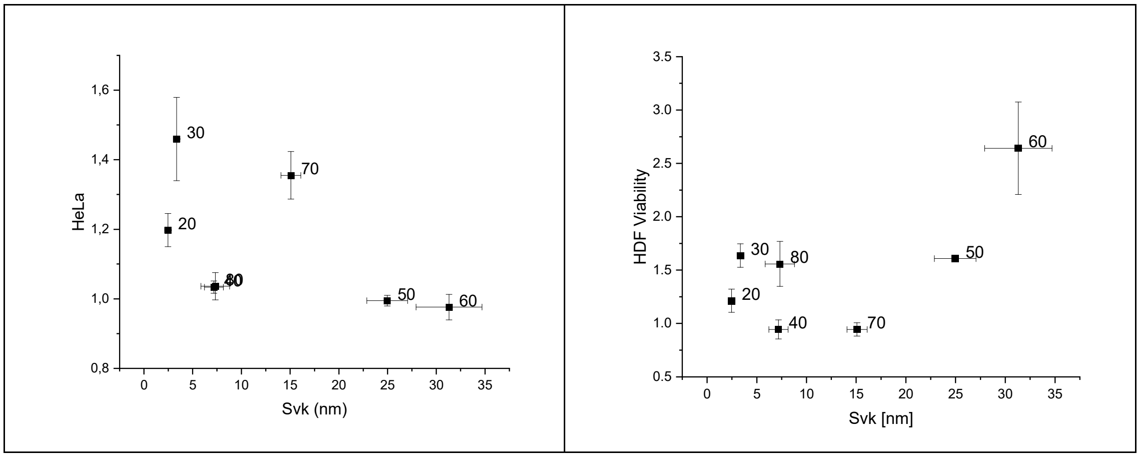

Supplementary Figure S9— The relative Hela cell yield (left) respectively relative HDF cell yield vs the S_{vk} parameter.

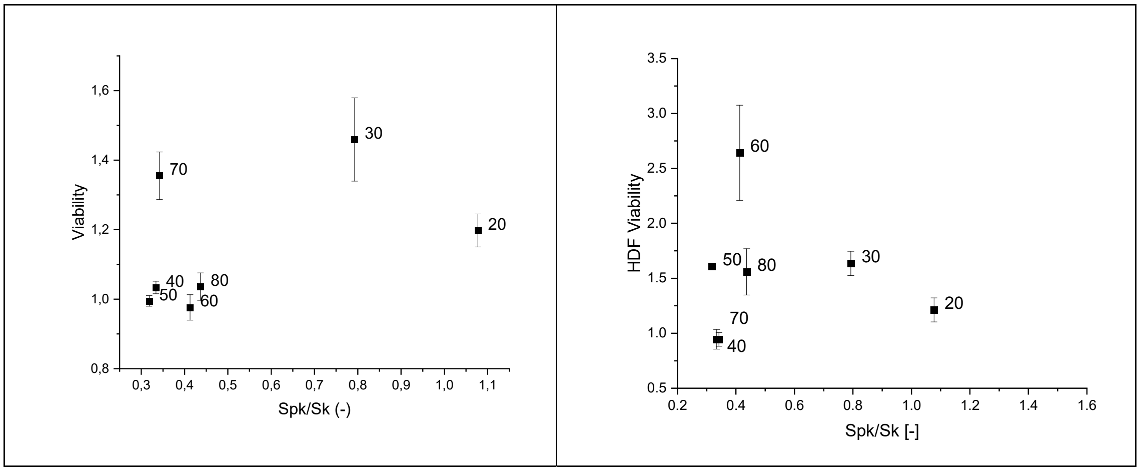

Supplementary Figure S10— The relative Hela cell yield (left) respectively relative HDF cell yield vs the S_{pk}/S_k parameter.

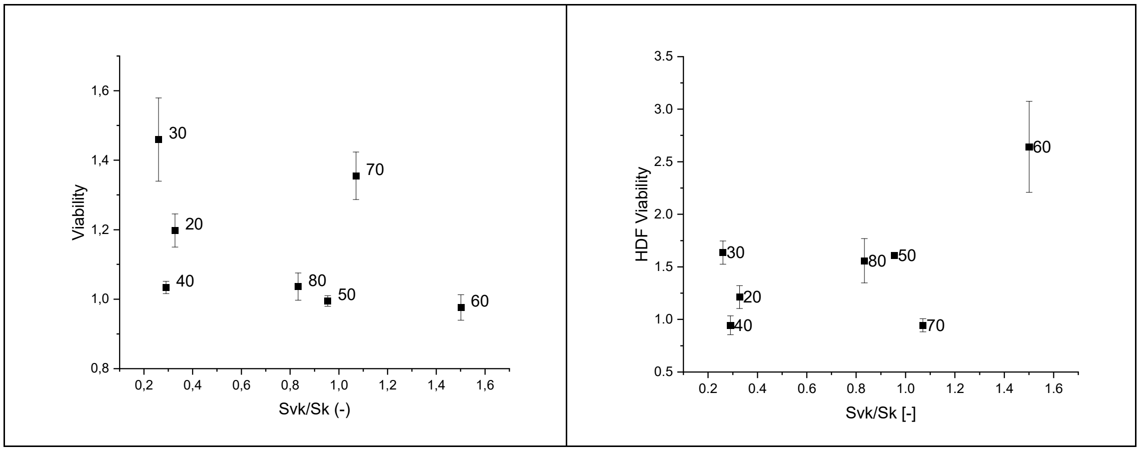

Supplementary Figure S11— The relative Hela cell yield (left) respectively relative HDF cell yield vs the S_{vk}/S_k parameter.

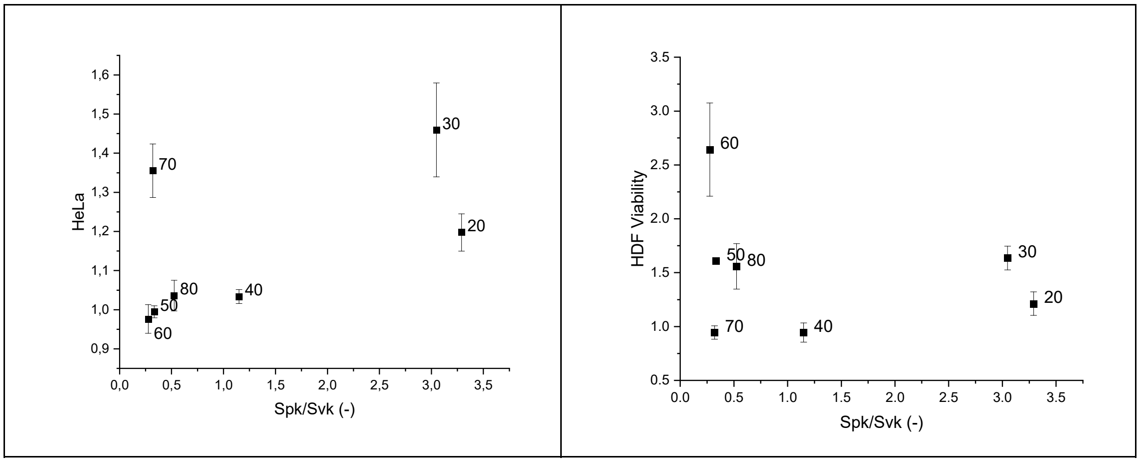

Supplementary Figure S12— The relative Hela cell yield (left) respectively relative HDF cell yield vs the S_{pk}/S_{vk} parameter.

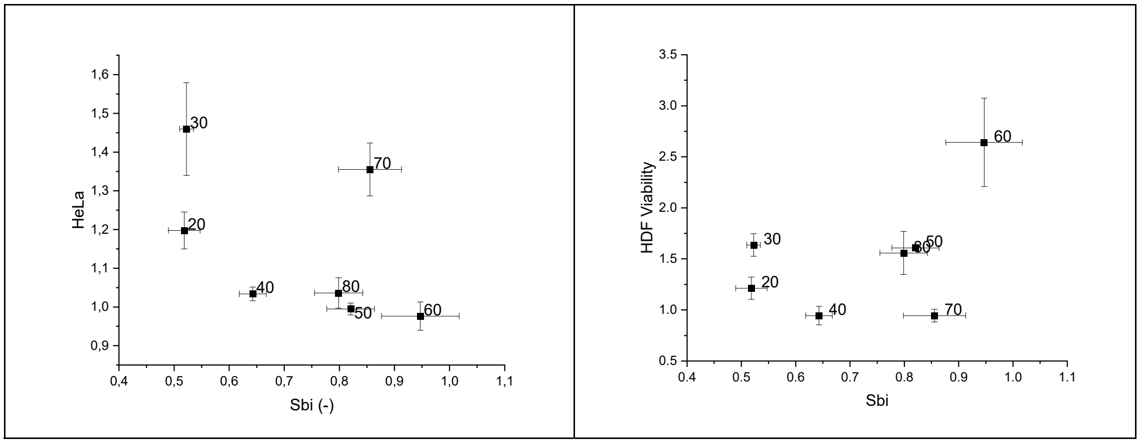

Supplementary Figure S13— The relative Hela cell yield (left) respectively relative HDF cell yield vs the S_{bi} parameter.

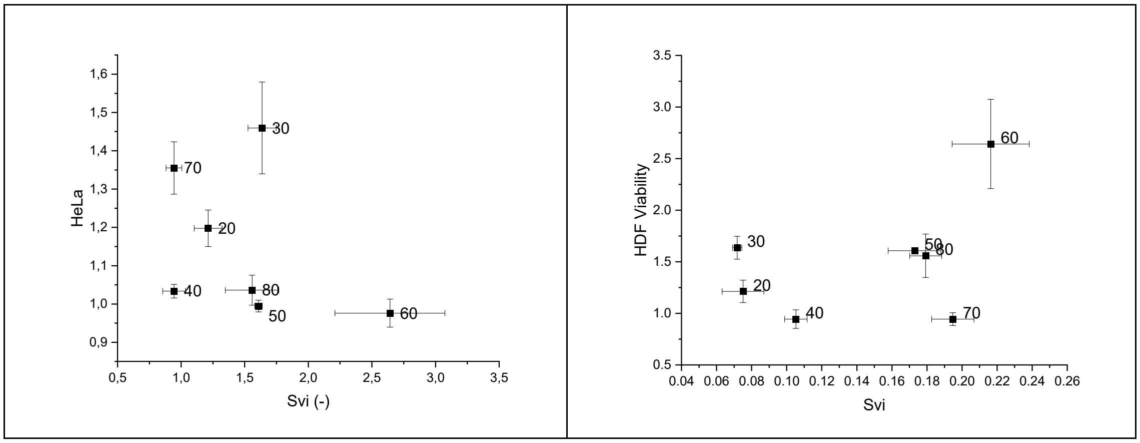

Supplementary Figure S14— The relative Hela cell yield (left) respectively relative HDF cell yield vs the S_{vi} parameter.

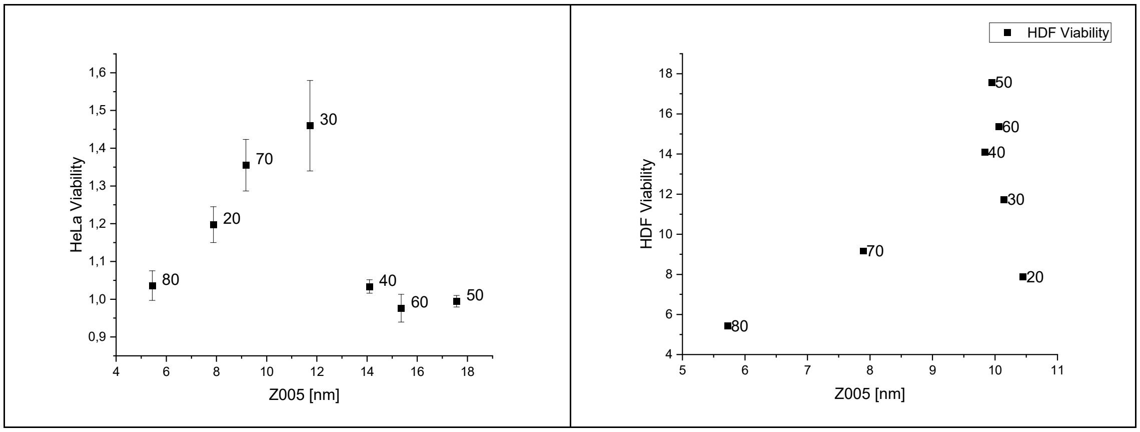

Supplementary Figure S15— The relative Hela cell yield (left) respectively relative HDF cell yield vs the $Z_{0.05}$ parameter.

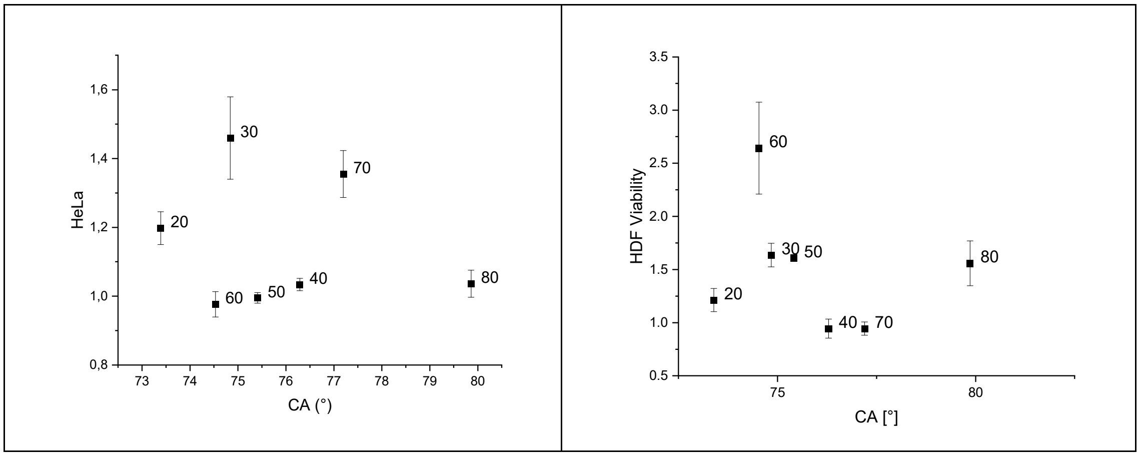

Supplementary Figure S16 — The relative Hela cell yield (left) respectively relative HDF cell yield vs the water contact angle.

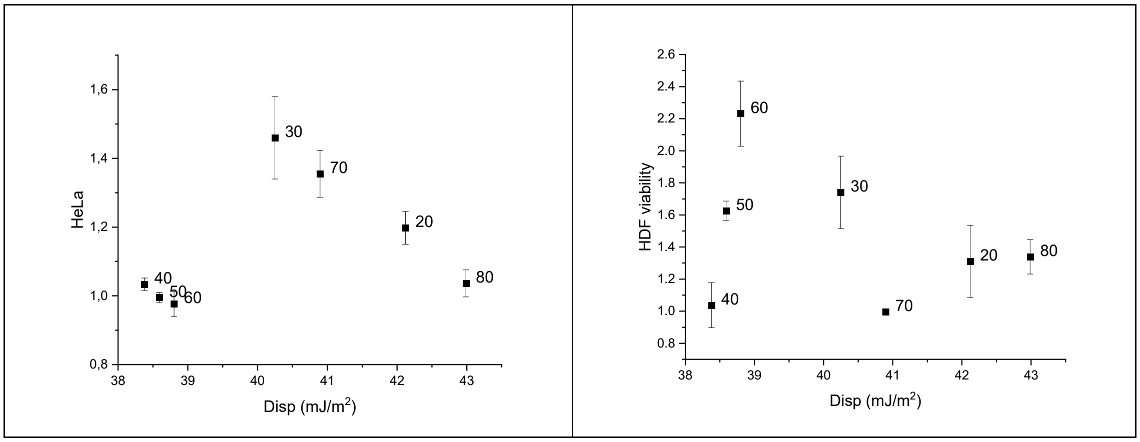

Supplementary Figure S17— The relative Hela cell yield (left) respectively relative HDF cell yield vs the dispersive surface energy component.

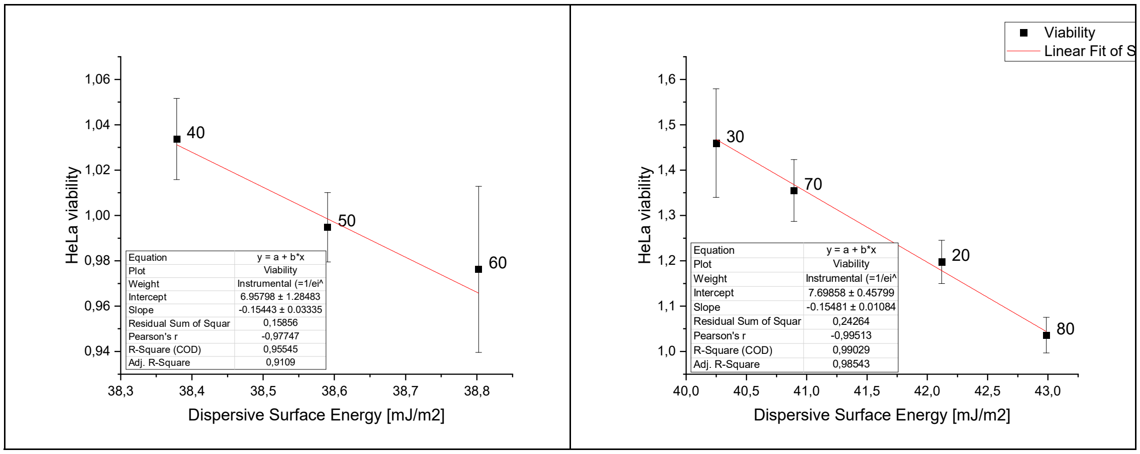

Supplementary Figure S18 — Linear fits for the two groupings found in the plot of the viability against the dispersive energy of the surfaces: right, the rougher surfaces (40%, 50%, and 60% PS) and left, the rest.

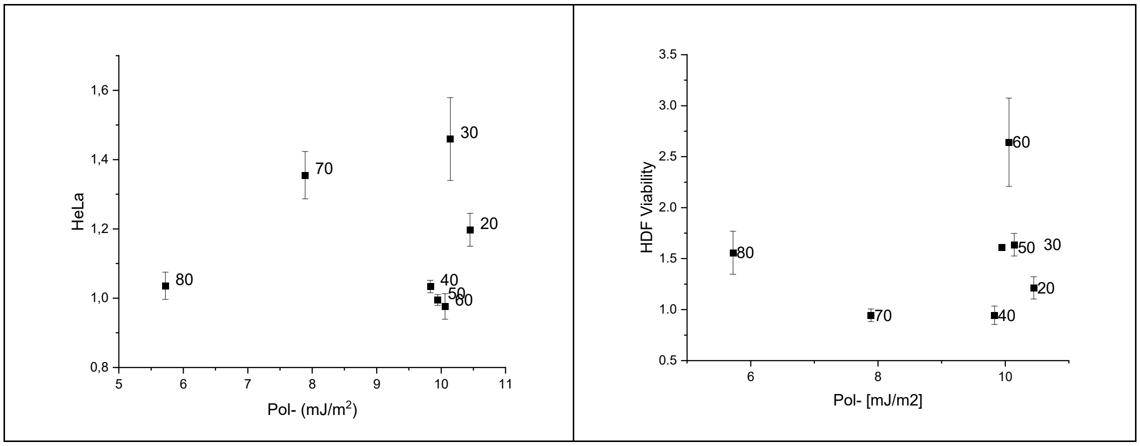

Supplementary Figure S19— The relative Hela cell yield (left) respectively relative HDF cell yield vs the polar negative surface energy component.

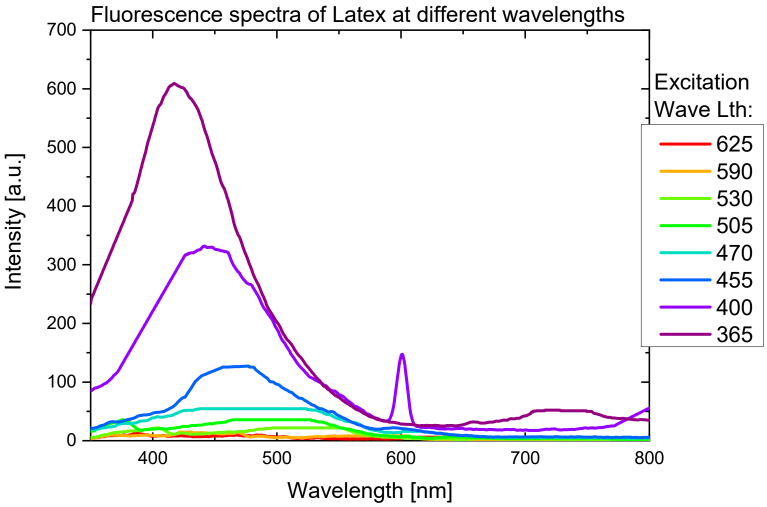

Supplementary Figure S20 — Fluorescence spectra of 50% PS lates showing its intrinsic autofluorescence.

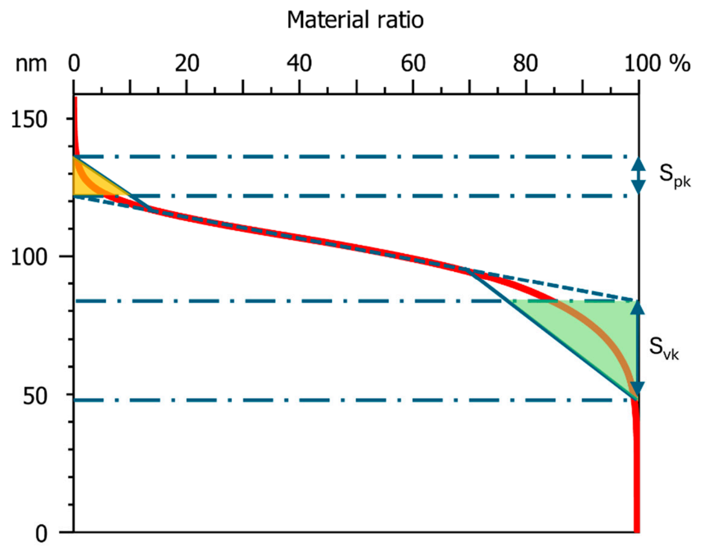

Supplementary Figure S21 — Illustration of the material ratio curve of a 5 µm x 5 µm images of 50% PS latex with marked S_{pk} and S_{vk} definitions.

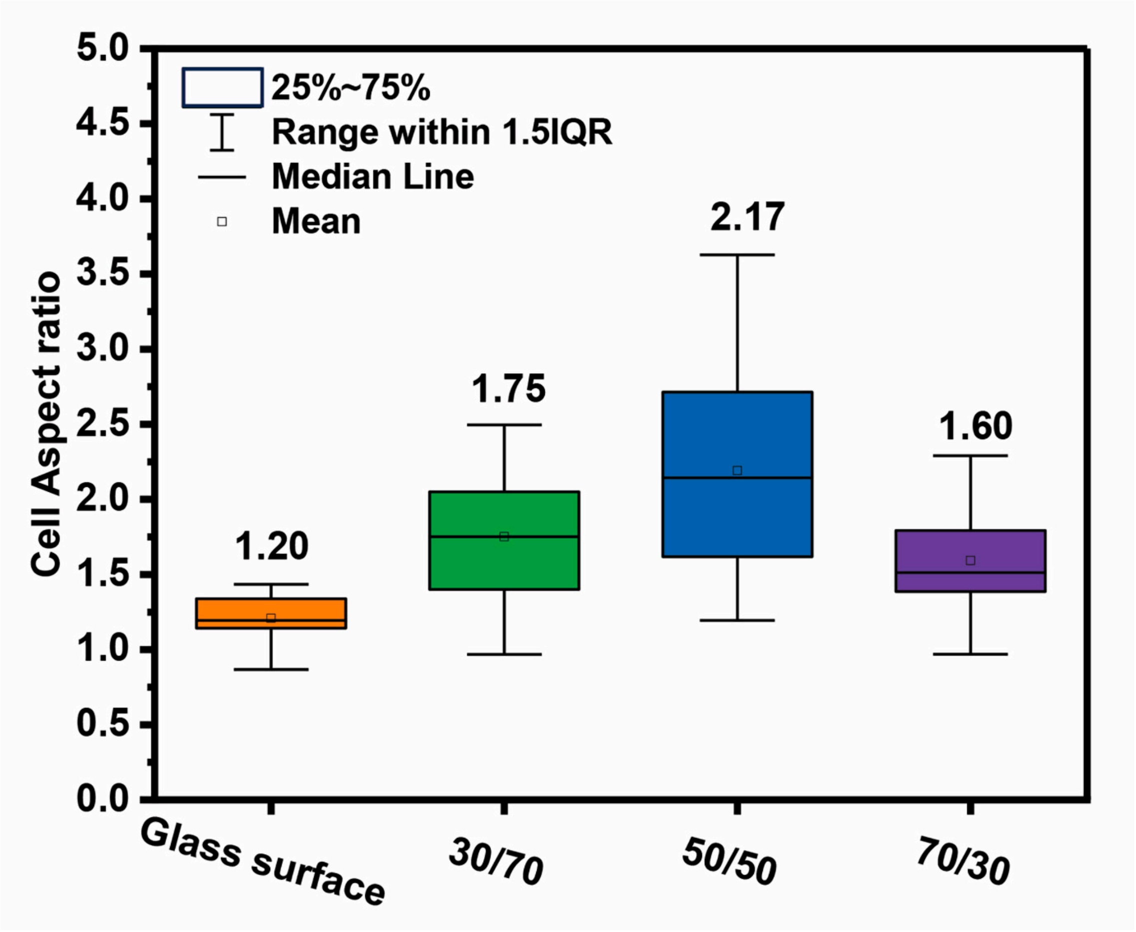

Supplementary Figure S22 — Aspect ratio (length/width) of the Live Hela cells on different surfaces after 96 h incubation.
